# Synthesis of a rhodium(iii) dinitrogen complex using a calix[4]arene-based diphosphine ligand†

**DOI:** 10.1039/d2cc06837k

**Published:** 2023-01-23

**Authors:** Jack Emerson-King, Sudip Pan, Matthew R. Gyton, Ralf Tonner-Zech, Adrian B. Chaplin

**Affiliations:** a Department of Chemistry, University of Warwick Gibbet Hill Road Coventry CV4 7AL UK a.b.chaplin@warwick.ac.uk; b Wilhelm-Ostwald-Institut für Physikalische und Theoretische Chemie, Universität Leipzig Linnéstraße 2 Leipzig D-04103 Germany

## Abstract

The synthesis and characterisation of the rhodium(iii) dinitrogen complex [Rh(2,2′-biphenyl)(CxP_2_)(N_2_)]^+^ are described, where CxP_2_ is a *trans*-spanning calix[4]arene-based diphosphine and the dinitrogen ligand is projected into the cavity of the macrocycle.

## Abstract 2

Capturing dinitrogen at a high-valent metal centre using a cavitand-based ligand.

## Graphical abstract

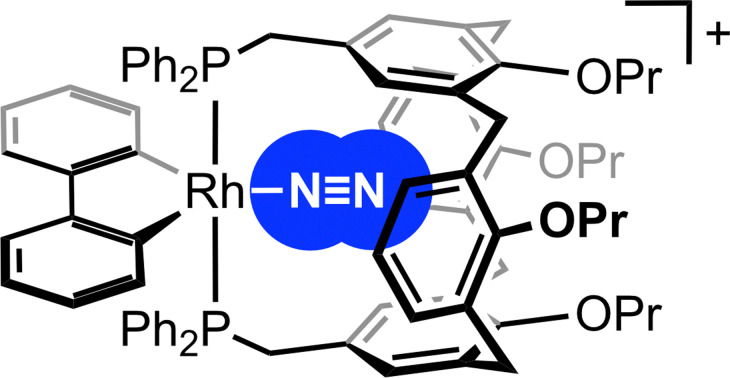

Activation of dinitrogen by coordination to a transition metal is a process of immense biological and technological importance, helping to weaken the otherwise formidably strong nitrogen–nitrogen triple bond (*D*_e_ = 946 kJ mol^−1^) through M→N_2_ π-back donation.^1,2^ Molecular dinitrogen complexes have been reported for most of the transition elements. Mononuclear d^6^ systems have been the most heavily investigated, however, no well-defined rhodium(iii) examples have previously been described.^3^ This paucity presumably reflects an incompatibility between the weak σ-donating, poor π-accepting character of dinitrogen and the relatively high oxidation state of the second-row transition metal.

Inspired by the use of donor-functionalised cavitands as ligands in the literature and as part of our work exploring the chemistry of low-coordinate group 9 complexes supported by the high trans influence ancillary ligand 2,2′-biphenyl (biph),^4,5^ we became intrigued by the prospect of using a cavitand-based ligand to isolate a labile rhodium(iii) dinitrogen complex.^6^ To this end, synthesis of [Rh(biph)(CxP_2_)(N_2_)][Al(OR^F^)_4_] (1-N_2_, R^F^ = C(CF_3_)_3_; Fig. 1A) was targeted, reasoning that the previously reported diphosphine ligand CxP_2_ would position the {Rh(biph)}^+^ fragment across the upper rim of the constituent calix[4]arene scaffold and in doing so favour coordination of the small diatomic over solvent molecules. Only polynuclear and dinuclear derivatives of CxP_2_ have been reported previously.^7^ We herein describe the synthesis and characterisation of 1-N_2_ through dehydration of the corresponding rhodium(iii) aqua complex 1-OH_2_, which can be obtained in 44% isolated yield by ligand substitution of *trans*-[Rh(biph)(PPh_3_)_2_(OH_2_)][Al(OR^F^)_4_] (2-OH_2_) with CxP_2_ in THF at room temperature (Fig. 1A).

**Fig. 1:**
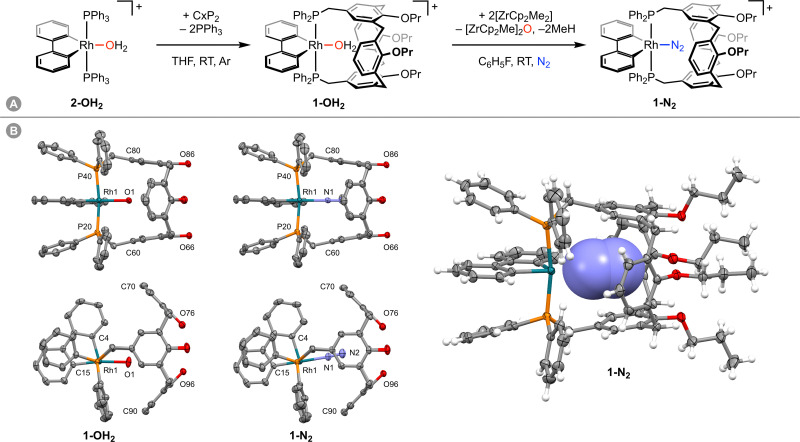
(A) Synthesis of 1-N_2_. [Al(OR^F^)_4_]^−^ counterions omitted. (B) Solid-state structures of 1-OH_2_ and 1-N_2_ with thermal ellipsoids at 50% probability; solvents, and anions omitted. Two perspective views shown for each complex without H atoms and Pr groups, with a third for 1-N_2_ showing encapsulated N_2_ in space fill with minor disordered components omitted (2×Pr). Selected bond lengths (Å) and angles (°): 1-OH_2_, Rh1–O1, 2.2046(14); Rh1–C4, 1.982(2); Rh1–C15, 1.996(2); C15–Rh1–O1, 172.17(7); Rh1–P20, 2.3505(5); Rh1–P40, 2.3403(5); P20–Rh1–P40, 171.90(2); shortest Rh1–C(phenyl), 3.485(2); 1-N_2_, Rh1–N2, 2.160(2); N1–N2, 1.091(3); Rh1–N1–N2, 176.7(2); Rh1–C4, 2.000(2); Rh1–C15, 2.009(2); C15–Rh1–N1, 179.43(10); Rh1–P20, 2.3732(6); Rh1–P40, 2.3630(6); P20–Rh1–P40, 169.79(2); shortest Rh1–C(phenyl), 3.470(3).

In CD_2_Cl_2_ solution, isolated 1-OH_2_ is characterised by time-averaged *C*_2v_ symmetry and a doublet ^31^P resonance at *δ* 13.2 (^1^*J*_RhP_ = 120 Hz) at 298 K. Coordination of water within the calix[4]arene cavity is evidenced by a singlet 2H resonance at *δ* 0.84, which was washed out upon shaking with D_2_O and is significantly shielded relative to free water (*δ* 1.53) and 2-OH_2_ (*δ* 2.44). Crystals of 1-OH_2_ suitable for analysis by single crystal X-ray diffraction were obtained from CH_2_Cl_2_-hexane and demonstrate that the metal adopts a square pyramidal coordination geometry with the CxP_2_ ligand bound with near ideal trans geometry (P20–Rh1–P40 = 171.90(2)°) in the solid state (Fig. 1B). The coordinated water ligand is projected into the calix[4]arene cavity with a Rh1–O1 bond distance of 2.2046(14) Å and approximately linear C15–Rh1–O1 angle of 172.17(8)°. The formally vacant coordination site of the metal centre is sterically occluded by two phenyl groups of the CxP_2_ ligand, with carbon contacts >3.4 Å suggesting that any stabilisation by agostic bonding is minimal.^8^ In any case, these phenyl groups complete the encapsulation of the aqua ligand, which is contained within an almost uninterrupted van der Waals surface defined by the components of 1.

Treatment of 1-OH_2_ with an excess of the potent drying agent [ZrCp_2_Me_2_]^9^ in CD_2_Cl_2_ under an atmosphere of dinitrogen resulted in smooth conversion into a 6 : 5 dynamic equilibrium mixture of new rhodium(iii) CxP_2_ complexes we assign as 1-N_2_ (*δ*_31P_ 16.1, ^1^*J*_RhP_ = 117 Hz) and 1-DCM (*δ*_31P_ 4.4, ^1^*J*_RhP_ = 117 Hz) within 24 h at room temperature. These assignments were substantiated by freeze-pump-thaw degassing the solution to remove dinitrogen and a control reaction carried out under an atmosphere of argon, both of which resulted in exclusive formation of 1-DCM. Highlighting the decisive role of the calix[4]arene scaffold, the spectroscopic characteristics of the bis(triphenylphosphine) analogue *trans*-[Rh(biph)(PPh_3_)_2_(κ^1^-ClCH_2_Cl)][Al(OR^F^)_4_] (2-DCM) are unchanged under an atmosphere of dinitrogen. Encouraged by these findings, the reaction between 1-OH_2_ and [ZrCp_2_Me_2_] was repeated under an atmosphere of dinitrogen in the more weakly coordinating solvent fluorobenzene.^10^ Consistent with our interpretation so far, 1-N_2_ (*δ*_31P_ 16.0; ^1^*J*_RhP_ = 116 Hz) was the only dehydration product observed by NMR spectroscopy.^11^ Subsequent analysis of 1-N_2_ by solution-phase IR spectroscopy provided direct evidence for coordination of dinitrogen. A very low intensity signal was observed at 2290 cm^−1^ and is tentatively assigned to the *ν*(N≡N) band. This band is red-shifted relative to free N_2_ (2330 cm^−1^), but considerably higher frequency than previously reported for terminal group 9 examples (1910–2236 cm^−1^).^1,3^ The assignment is supported by exposure of the sample to air, which resulted in disappearance of the *ν*(N≡N) band and formation of 1-OH_2_ within 5 seconds, slow evaporation of the solvent and analysis of the residue by ATR-IR spectroscopy within a dinitrogen filled glovebox, and computational analysis.

Despite numerous attempts, our efforts to isolate analytically pure samples of 1-N_2_ from solution were frustrated by the extremely strong affinity of 1 for water, resulting in contamination of samples with 1-OH_2_ by reaction with adventurous water.^12^ In one instance we were, however, able to obtain a single crystal of 1-N_2_ suitable for analysis by X-ray diffraction, by slow diffusion of hexane into a CH_2_Cl_2_ solution of 1-N_2_ generated *in situ* using [ZrCp_2_Me_2_] (Fig. 1B). The dinitrogen ligand was readily located from the Fourier difference map, was freely refined with 100% crystallographic occupancy, and there is no evidence for significant disorder (Fourier peaks <0.5 e Å^−3^). Whilst this crystal was not representative of the bulk of the sample, it is the first structurally characterised example of a rhodium(iii) dinitrogen complex. The solid-state structure of 1-N_2_ is isomorphous to 1-OH_2_ and the bulk geometric features of the {Rh(biph)(CxP_2_)}^+^ fragment are consequently similar. There are, however, statistically significant perturbations to the metal-centred metrics. For instance, the Rh1–P20 (2.3732(6) *vs.* 2.3505(5) Å) and Rh1–P40 (2.3630(6) *vs.* 2.3403(5) Å) bonds are elongated in 1-N_2_, whilst the P20–Rh1–P40 bond angle is contracted (169.79(2) *vs.* 171.90(2)°) relative to 1-OH_2_. Coordination of dinitrogen is also associated with a straighter C15–Rh1–N1 angle (179.43(10)°) than the corresponding metric in 1-OH_2_ (172.17(7)°), presumably to accommodate the linear diatomic within the calix[4]arene cavity. Both terminal and bridging end-on rhodium(i) dinitrogen complexes have been structurally characterised in the solid-state by X-ray diffraction, with the corresponding Rh–N bond lengths ranging from 1.85 to 2.08 Å (CSD *version* 5.43).^13^ Consistent with weak binding to the higher metal oxidation state, the Rh1–N1 bond length observed in 1-N_2_ is substantially longer than all these examples (2.160(2) Å). As for the aqua derivative, 1-N_2_ is fluxional in solution on the NMR time scale, adopting time-averaged *C*_2v_ symmetry in solution at 298 K. We attempted to probe coordination of dinitrogen by ^15^N NMR spectroscopy using an isotopically enriched sample in fluorobenzene, but only free dinitrogen was observed. Presumably ligand exchange is fast on the timescale of the NMR experiment at 298 K.

To help delineate the role of the calix[4]arene scaffold, a DFT-based energy decomposition analysis was performed in combination with natural orbitals for chemical valence (EDA-NOCV; PBE-D3(BJ)/TZ2P-ZORA level of theory) using minimum energy structures of 1-L and 2-L (L = H_2_O, N_2_, DCM; optimised at the PBE-D3(BJ)/def2-SVP level of theory).^14^ Consistent with our hypothesis that CxP_2_ destabilises solvent over dinitrogen coordination, the calculated bond dissociation energies (*D*_e_/kJ mol^−1^) decrease in the order H_2_O (89.6) > DCM (71.3) > N_2_ (68.6) for the bis(triphenylphosphine) complexes 2-L, but H_2_O (118.9) > N_2_ (81.9) ≫ DCM (33.9) for 1-L. Dichloromethane is not only too large to be accommodated within the calix[4]arene scaffold in 1, but requires a destabilising conformational change to permit metal coordination adjacent to the upper rim of the macrocycle (Δ*E*_prep_ = +50.7, *cf.* +16.4 kJ mol^−1^ for 2). The interaction between 1 and dinitrogen is characterised by a greater extent of σ-donation (Δ*E*_L→M_ = 48.9%) than π-back bonding (Δ*E*_M→L_ = 44.6%) and no meaningful covalent interactions with the cavity were identified from the NOCV analysis (Fig. S38, ESI†). This net charge transfer to the metal is unusual for dinitrogen complexes observed in the condensed phase, but in line with the high N≡N stretching frequency measured.^15^

In summary, structural and spectroscopic characterisation of a well-defined rhodium(iii) dinitrogen complex is reported. This complex is notable for a remarkably long rhodium–nitrogen bond (2.160(2) Å), a high N≡N vibrational band (2290 cm^−1^), and showcases the utility of donor-functionalised cavitands for interrogating small molecular activation reactions mediated by transition metals.

We thank Andy Ashley (Imperial College London) for supply of ^15^N_2_. We gratefully acknowledge the EPSRC (DTA studentship to J. E.-K.), European Research Council (ERC, grant agreement 637313; M. R. G.), and Royal Society (UF100592, UF150675, A. B. C.) for financial support. Crystallographic data were collected using an instrument that received funding from the ERC under the European Union's Horizon 2020 research and innovation programme (grant agreement no. 637313). Computational resources were provided by ZIH Dresden and GOETHE-CSC Frankfurt.

## Conflicts of interest

The authors declare no conflicts of interest.

## Supplementary Material

CC-059-D2CC06837K-s001

CC-059-D2CC06837K-s002

CC-059-D2CC06837K-s003
