# Supplementary material for: Synthesis of a rhodium(iii) dinitrogen complex using a calix[4]arene-based diphosphine ligand
Source: Chem Commun (Camb). 2023 Jan 23;59(15):2150–2. doi: 10.1039/d2cc06837k (PMC9933454; doi:10.1039/d2cc06837k)
Supplement: CC-059-D2CC06837K-s001 [file CC-059-D2CC06837K-s001.pdf]

Electronic Supplementary Material (ESI) for Chemical Communications.
This journal is © The Royal Society of Chemistry 2023

Supporting information for:

Synthesis of a rhodium(III) dinitrogen complex using a calix[4]arene-based diphosphine ligand

Jack Emerson-King, Sudip Pan, Matthew R. Gyton, Ralf Tonner-Zech, and Adrian B. Chaplin.

Contents

1. General experimental methods. 2
2. Synthesis of CxP_2 3
  2.1. Preparation of 25,26,27,28-tetrahydroxycalix[4]arene (**C1**) 3
  2.2. Preparation of 25,26,27,28-tetrapropoxycalix[4]arene (**C2**) 4
  2.3. Preparation of 5,11,17,23-tetrabromo-25,26,27,28-tetrapropoxycalix[4]arene (**C3**) 5
  2.4. Preparation of 5,17-dibromo-25,26,27,28-tetrapropoxycalix[4]arene (**C4**) 5
  2.5. Preparation of 5,17-diformyl-25,26,27,28-tetrapropoxycalix[4]arene (**C5**) 6
  2.6. Preparation of 5,17-bis(hydroxymethyl)-25,26,27,28-tetrapropoxycalix[4]arene (**C6**) 7
  2.7. Preparation of 5,17-bis(chloromethyl)-25,26,27,28-tetrapropoxycalix[4]arene (**C7**) 7
  2.8. Preparation of 5,17-bis(bromomethyl)-25,26,27,28-tetrapropoxycalix[4]arene (**C8**) 8
  2.9. Preparation of CxP_2 9
3. Synthesis and reactivity of rhodium triphenylphosphine complexes 10
  3.1. Preparation of *trans*-$[Rh(biph)(PPh_3)_2(\kappa^1\text{-}ClCH_2Cl)][Al(OR^F)_4]$ (**2-DCM**) 10
  3.2. Stability of **2-DCM** under dinitrogen. 12
  3.3. Preparation of *trans*-$[Rh(biph)(PPh_3)_2(OH_2)][Al(OR^F)_4]$ (**2-OH₂**) 13
4. Synthesis and reactivity of rhodium CxP_2 complexes 15
  4.1. Preparation of $[Rh(biph)(CxP_2)(OH_2)][Al(OR^F)_4]$ (**1-OH₂**) 15
  4.2. Reaction of **1-OH₂** with D_2O 18
  4.3. Dehydration of **1-OH₂** using $[ZrCp_2Me_2]$ in dichloromethane under dinitrogen 19
  4.4. Dehydration of **1-OH₂** using $[ZrCp_2Me_2]$ in fluorobenzene under dinitrogen 20
  4.5. Analysis of **1-N₂** by ^{15}N NMR spectroscopy 21
  4.6. Analysis of **1-N₂** by IR spectroscopy 22
5. Crystallography 24
6. Computational details 24
7. References 28

1. General experimental methods.

All manipulations were performed under argon using standard Schlenk line and glove box techniques unless otherwise stated. Glassware was oven dried at 150 °C overnight and flamed under vacuum prior to use. 3 Å molecular sieves were activated by heating at 300 °C *in vacuo* overnight prior to use. Anhydrous solvents were obtained from commercial sources. Hexane was further dried over Na/K_2 alloy, vacuum-distilled, and freeze-pump-thaw degassed before being placed under argon over a potassium mirror. CH_2Cl_2 was further dried over CaH_2 overnight, vacuum-distilled, and freeze-pump-thaw degassed three times before being placed under argon over activated 3 Å molecular sieves. THF was further dried over Na/benzophenone, vacuum-distilled, and freeze-pump-thaw degassed three times before being placed under argon over activated 3 Å molecular sieves. Fluorobenzene was stirred over neutral alumina, filtered, stirred over CaH_2 overnight, vacuum-distilled, and freeze–pump–thaw degassed three times before being placed under argon over activated 3 Å molecular sieves.[1] CD_2Cl_2 was placed over activated 3 Å molecular sieves and freeze-pump-thaw degassed three times before being placed under argon. *trans*-[Rh(biph)(PPh_3)$_2$Cl][2,3] and CxP_2[4] were prepared using literature procedures, or minor variations thereof. For convenience, the full multi-step procedure for the latter is reported below. All other reagents and solvents are commercial products and were used as received. NMR spectra were recorded on Bruker spectrometers at 298 K unless otherwise stated. Chemical shifts are quoted in ppm and coupling constants in Hz. Virtual coupling constants are reported as the separation between the first and third lines.[5] ^{1}H and ^{31}P{^{1}H} NMR spectra recorded in fluorobenzene were referenced using an internal sealed capillary of a 25 mM solution of trimethylphosphate in C_6D_6 (δ_{1H} 3.38, $^3J_{PH}$ = 11 Hz; δ_{31P} = 3.7).[6] The <u>C</u>(CF_3)$_3$ carbon resonance of the [Al(OR^F)$_4$]$^-$ anion was not observed in any ^{13}C{^{1}H} NMR spectra described herein. High-resolution ESI mass spectra (HR ESI-MS) were recorded on a Bruker Maxis Plus instrument. Low-resolution ESI mass spectra (LR ESI-MS) were recorded on an Agilent 6130B single Quad instrument. Solution-phase IR spectroscopy was performed on a PerkinElmer Spectrum 100 spectrometer. ATR-IR spectroscopy was performed on a Bruker ALPHA FT-IR spectrometer. Microanalyses were performed by Stephen Boyer at London Metropolitan University.

2. Synthesis of CxP_2

5,17-bis(diphenylphospinomethy)-25,26,27,28-tetrapropoxycalix[4]arene (CxP_2) was synthesised over eight steps in 17% overall yield from commercial *tert*-butylcalix[4]arene using modified literature protocols which are summarised in Figure S1. In the final step, the diphosphine can be accessed from bis(bromomethyl) or bis(chloromethyl) functionalised calix[4]arenes (**C7** and **C8**) with $KPPh_2$, but the former is higher yielding.

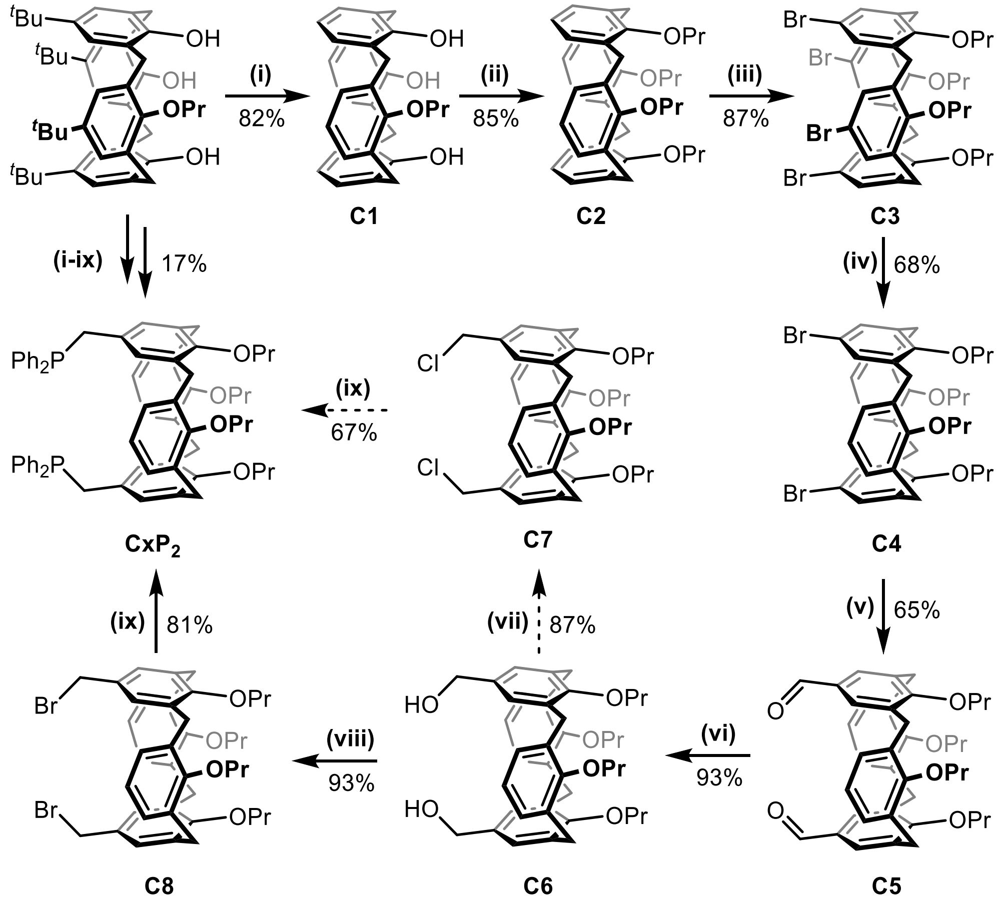

Figure S1. Synthesis of CxP_2: (i) $AlCl_3$, PhOH, toluene, RT, 5 h; (ii) PrI, NaH, DMF, 80 °C 18 h; (iii) N-bromosuccinimide, THF, RT, 24 h; (iv) ^{n}BuLi, THF, -78 °C, 20 min; MeOH; (v) ^{n}BuLi, THF, -78 °C, 15 min; DMF; (vi) $NaBH_4$, EtOH, 1.5 h; (vii) $SOCl_2$, CH_2Cl_2, -78 °C to RT, 2.5 h; (viii) PBr_3, CH_2Cl_2, RT, 10 min; (ix) $KPPh_2$, THF, -78 to RT, 18 h.

2.1. Preparation of 25,26,27,28-tetrahydroxycalix[4]arene (C1)

Under N_2: a suspension of *tert*-butylcalix[4]arene (30.0 g, 46.2 mmol), $AlCl_3$ (46.3 g, 347 mmol) and phenol (21.8 g, 231 mmol) in toluene (1 L) was stirred at ambient temperature for 5 h. In air: the reaction mixture was poured onto $HCl_{(aq)}$ (2 M, 800 mL) and then extracted into CH_2Cl_2 (800 mL). The organic phase was washed with H_2O (3×400 mL), dried over $MgSO_4$, and the solvent removed *in vacuo*. The residue was dissolved in CH_2Cl_2 (50 mL) and the product precipitated by the addition of MeOH (400 mL), isolated by filtration, and dried *in vacuo*. Yield: 16.0 g (37.7 mmol, 82%; white solid). Spectroscopic data are consistent with previous reports.[7]

^{1}H NMR ($CDCl_3$, 400 MHz): δ 10.19 (s, 4H, OH), 7.05 (d, $^3J_{HH}$ = 7.6, 8H, *m*-Ar), 6.73 (t, $^3J_{HH}$ = 7.6, 4H, *p*-Ar), 4.26 (br, 4H, ArC<u>H</u>$_2$Ar), 3.55 (br, 4H, ArC<u>H</u>$_2$Ar).
LR ESI-MS (negative ion): 423.2 ([*M*–H]$^-$, calcd 423.2) *m*/*z*.

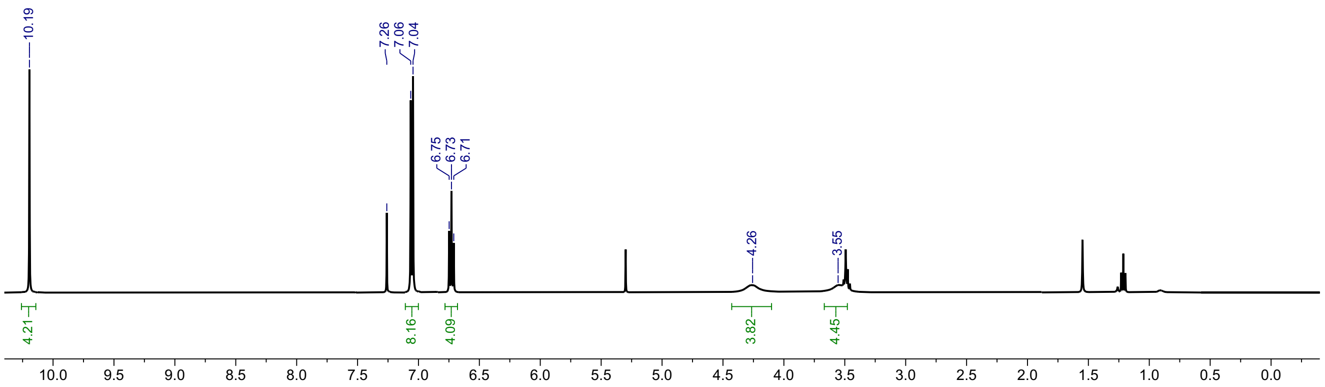

Figure S2. ^{1}H NMR spectrum of **C1** ($CDCl_3$, 400 MHz).

2.2. Preparation of 25,26,27,28-tetrapropoxycalix[4]arene (C2)

Under N_2: to a solution of **C1** (14.9 g, 35.0 mmol) in DMF (1 L) was added PrI (29.4 mL, 302 mmol) and NaH (10.9 g, 452 mmol) and the reaction stirred at 80 °C for 18 h. In air: the suspension was cooled to ambient temperature, poured onto H_2O (1 L), and then extracted into CH_2Cl_2 (1 L). The organic phase was washed with $HCl_{(aq)}$ (2 M, 2×1 L), dried over $MgSO_4$, and the solvent removed *in vacuo*. The residue was dissolved in CH_2Cl_2 (50 mL) and the product precipitated by the addition of MeOH (400 mL), isolated by filtration, and dried *in vacuo*. Yield: 17.6 g (29.7 mmol, 79%; white solid). Spectroscopic data are consistent with previous reports.[8]

^{1}H NMR ($CDCl_3$, 300 MHz): δ 6.68 – 6.49 (m, 12H, *m*-Ar+*p*-Ar), 4.46 (d, $^2J_{HH}$ = 13.3, 4H, ArC<u>H</u>$_2$Ar), f3.85 (t, $^3J_{HH}$ = 7.5, 8H, OCH_2), 3.15 (d, $^2J_{HH}$ = 13.4, 4H, ArC<u>H</u>$_2$Ar), 1.93 (hex, $^3J_{HH}$ = 7.5, 8H, C<u>H</u>$_2$CH$_3$), 1.00 (t, $^3J_{HH}$ = 7.5, 12H, CH$_2$C<u>H</u>$_3$).
LR ESI-MS (positive ion): 615.5 ([*M*+Na]$^+$, calcd 615.3) *m*/*z*.

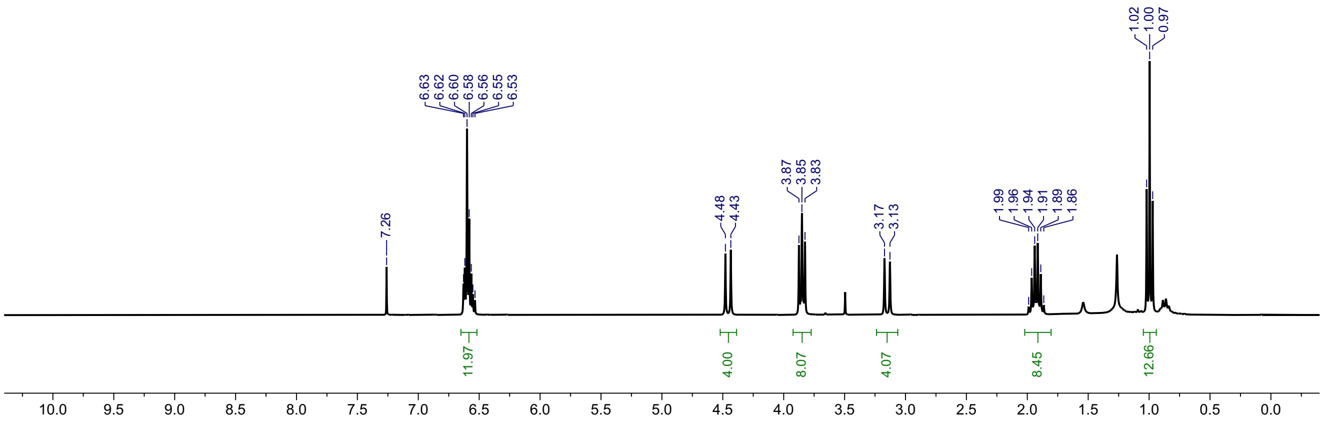

Figure S3. ^{1}H NMR spectrum of **C2** ($CDCl_3$, 300 MHz).

2.3. Preparation of 5,11,17,23-tetrabromo-25,26,27,28-tetrapropoxycalix[4]arene (C3)

Under N_2: a solution of **C2** (4.10 g, 6.92 mmol) and N-bromosuccinimide (11.1 g, 62.2 mmol) in THF (250 mL) was stirred at ambient temperature for 24 h. In air: the reaction mixture was poured onto $NaHSO_{3(aq)}$ (10 wt%, 250 mL) and then extracted into CH_2Cl_2 (250 mL). The organic phase was washed with H_2O (2×250 mL) and dried over $MgSO_4$. The solution was concentrated to 50 mL and the product precipitated by the addition of MeOH (400 mL), isolated by filtration, and dried *in vacuo*. Yield: 5.44 g (5.99 mmol, 87%; white solid). Spectroscopic data are consistent with previous reports.[9]

^{1}H NMR ($CDCl_3$, 300 MHz): δ 6.80 (s, 8H, *m*-Ar), 4.35 (d, 4H, $^2J_{HH}$ = 13.5, ArC<u>H</u>$_2$Ar), 3.80 (t, $^3J_{HH}$ = 7.5, 8H, OCH_2), 3.08 (d, 4H, $^2J_{HH}$ = 13.5, ArC<u>H</u>$_2$Ar), 1.87 (hex, $^3J_{HH}$ = 7.4, 8H, C<u>H</u>$_2$CH$_3$), 0.96 (t, $^3J_{HH}$ = 7.4, 12H, CH$_2$C<u>H</u>$_3$).

LR ESI-MS (positive ion): 931.1 ([*M*+Na]$^+$, calcd 931.0) *m*/*z*.

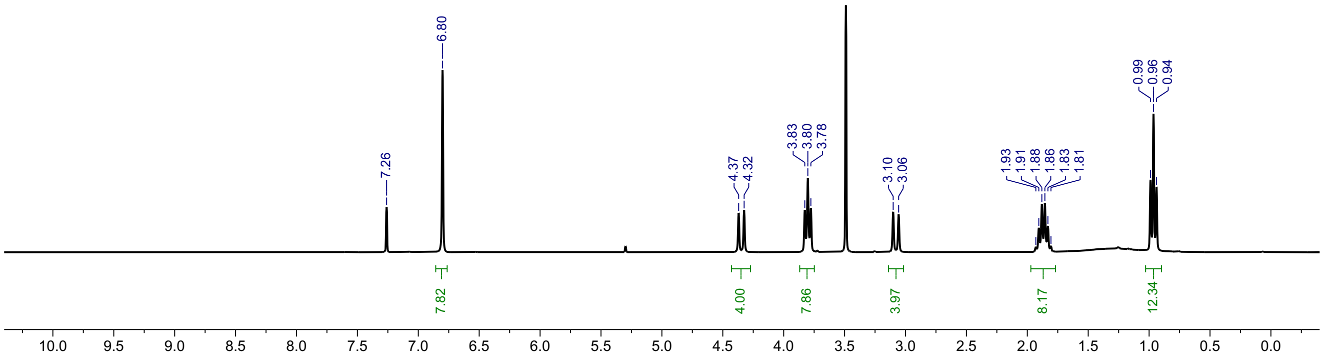

Figure S4. ^{1}H NMR spectrum of **C3** ($CDCl_3$, 300 MHz).

2.4. Preparation of 5,17-dibromo-25,26,27,28-tetrapropoxycalix[4]arene (C4)

Under N_2: to a solution of **C3** (22.0 g, 24.2 mmol) in THF (750 mL) cooled to -78 °C was added ^{n}BuLi (38.0 mL, 1.6 M in hexanes, 60.8 mmol) and the reaction stirred for 20 mins, then quenched with the addition of MeOH (100 mL). In air: the mixture was warmed to ambient temperature, poured onto ice-cold HCl(aq) (2 M, 750 mL), and extracted into CH_2Cl_2 (500 mL). The organic phase was washed with H_2O (2×500 mL), dried over $MgSO_4$, and the solvent removed *in vacuo.* The residue was dissolved in CH_2Cl_2 (50 mL) and the product precipitated by the addition of MeOH (400 mL), isolated by filtration, and dried *in vacuo*. Yield: 12.4 g (16.5 mmol, 68%; white solid). Spectroscopic data are consistent with previous reports.[9]

^{1}H NMR ($CDCl_3$, 300 MHz): δ 6.77 (s, 4H, *m*-Ar^{Br}), 6.64 (s, 6H, *m*-Ar+*p*-Ar), 4.40 (d, $^2J_{HH}$ = 13.4, 4H, ArC<u>H</u>$_2$Ar^{Br}), 3.84 (t, $^3J_{HH}$ = 6.9, 4H, OCH_2), 3.81 (t, $^3J_{HH}$ = 6.9, 4H, OCH_2), 3.11 (d, $^2J_{HH}$ = 13.4, 4H, ArC<u>H</u>$_2$Ar^{Br}), 1.91 (hex, $^3J_{HH}$ = 7.4, 8H, 2×C<u>H</u>$_2$CH$_3$), 0.99 (t, $^3J_{HH}$ = 7.5, 6H, CH$_2$C<u>H</u>$_3$), 0.98 (t, $^3J_{HH}$ = 7.5, 6H, CH$_2$C<u>H</u>$_3$).

LR ESI-MS (positive ion): 773.3 ([*M*+Na]$^+$, calcd 773.2) *m*/*z*.

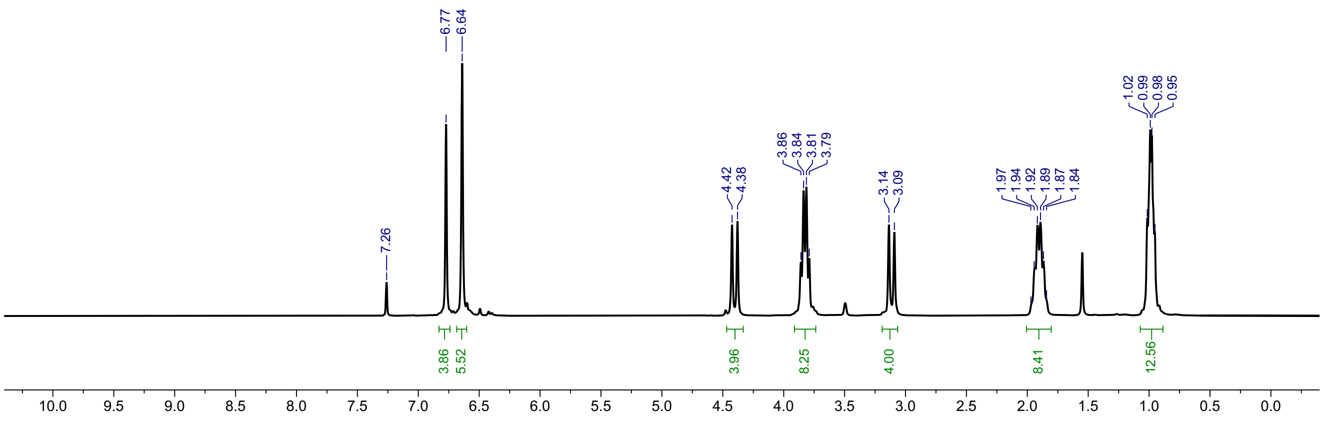

Figure S5. ^{1}H NMR spectrum of **C4** ($CDCl_3$, 300 MHz).

2.5. Preparation of 5,17-diformyl-25,26,27,28-tetrapropoxycalix[4]arene (C5)

Under N_2: to a solution of **C4** (11.4 g, 15.2 mmol) in THF (500 mL) cooled to -78 °C was added ^{n}BuLi (38.0 mL, 1.6 M in hexanes, 60.8 mmol) and the reaction stirred for 15 mins, then quenched with the addition of DMF (25 mL, 304 mmol). In air: the mixture was warmed to ambient temperature, poured onto ice-cold $HCl_{(aq)}$ (2 M, 500 mL), and extracted into CH_2Cl_2 (500 mL). The organic phase was washed with H_2O (2×500 mL), dried over $MgSO_4$, and the solvent removed *in vacuo*. The crude residue was dissolved in hot EtOH (100 mL) and cooled to 4 °C to precipitate the product, which was isolated by filtration and dried *in vacuo*. Yield: 6.40 g (9.86 mmol, 65%, white solid). Spectroscopic data are consistent with previous reports.[9]

^{1}H NMR ($CDCl_3$, 300 MHz): δ 9.47 (s, 2H, CHO), 7.00 (s, 4H, *m*-Ar^{CHO}), 6.85–6.61 (m, 6H, *m*-Ar+*p*-Ar), 4.47 (d, $^2J_{HH}$ = 13.6, 4H, ArC$\underline{H}_2$Ar^{CHO}), 4.01–3.68 (m, 8H, 2×OCH_2), 3.23 (d, $^2J_{HH}$ = 13.6, 4H, ArC$\underline{H}_2$Ar^{CHO}), 1.91 (hex, $^3J_{HH}$ = 7.4, 8H, 2×C$\underline{H}_2$$CH_3$), 1.03 (t, $^3J_{HH}$ = 7.3, 6H, CH_2C$\underline{H}_3$), 0.97 (t, $^3J_{HH}$ = 7.3, 6H, CH_2C$\underline{H}_3$).

LR ESI-MS (positive ion): 671.4 ([*M*+Na]$^+$, calcd 671.3) *m*/*z*.

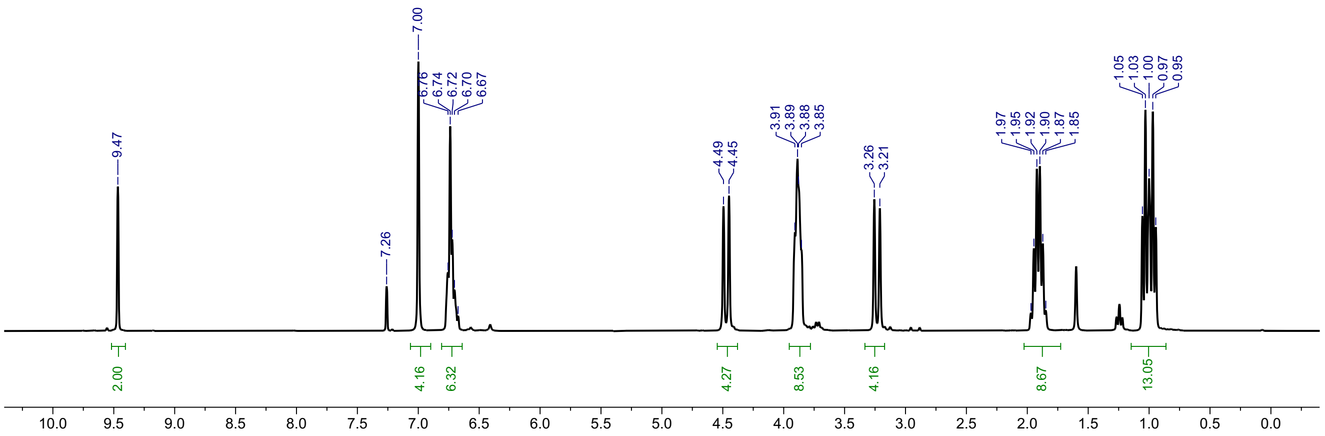

Figure S6. ^{1}H NMR spectrum of **C5** ($CDCl_3$, 300 MHz).

2.6. Preparation of 5,17-bis(hydroxymethyl)-25,26,27,28-tetrapropoxycalix[4]arene (C6)

Under N_2: to a solution of **C5** (6.48 g, 10.0 mmol) in EtOH (200 mL) was added $NaBH_4$ (0.378 g, 10 mmol) and the reaction stirred at ambient temperature for 1.5 h. In air: the solvent was removed in vacuo and the crude mixture extracted into CH_2Cl_2 (100 mL). The organic phase was washed with $HCl_{(aq)}$ (2 M, 2×100 mL), H_2O (2×100 mL), dried over $MgSO_4$, and the solvent removed *in vacuo*. The compound was purified by flash column chromatography (SiO_2; 2:2:3 CH_2Cl_2:EtOAc:hexane). Yield: 6.11 g (9.36 mmol, 93%; white solid). Spectroscopic data are consistent with previous reports.[10,11]

^{1}H NMR ($CDCl_3$, 300 MHz): δ 6.92 (d, $^3J_{HH}$ = 7.3, 4H, *m*-Ar), 6.79 (t, $^3J_{HH}$ = 7.2, 2H, *p*-Ar), 6.38 (s, 4H, *m*-Ar^{OH}), 4.46 (d, $^2J_{HH}$ = 13.2, 4H, $ArC\underline{H}_2Ar^{OH}$), 4.16 (s, 4H, $C\underline{H}_2OH$), 3.98 (t, $^3J_{HH}$ = 7.8, 4H, OCH_2), 3.73 (t, $^3J_{HH}$ = 6.8, 4H, OCH_2), 3.15 (d, $^2J_{HH}$ = 13.3, 4H, $ArC\underline{H}_2Ar^{OH}$), 1.98 (hex, $^3J_{HH}$ = 7.4, 4H, $C\underline{H}_2CH_3$), 1.90 (hex, $^3J_{HH}$ = 7.3, 4H, $C\underline{H}_2CH_3$), 1.80 (s, 2H, OH), 1.05 (t, $^3J_{HH}$ = 7.3, 6H, $CH_2C\underline{H}_3$), 0.94 (t, $^3J_{HH}$ = 7.4, 6H, $CH_2C\underline{H}_3$).

LR ESI-MS (positive ion): 675.5 ($[M+Na]^+$, calcd 675.4) *m*/*z*.

LR ESI-MS (negative ion): 651.4 ($[M–H]^-$, calcd 651.4) *m*/*z*.

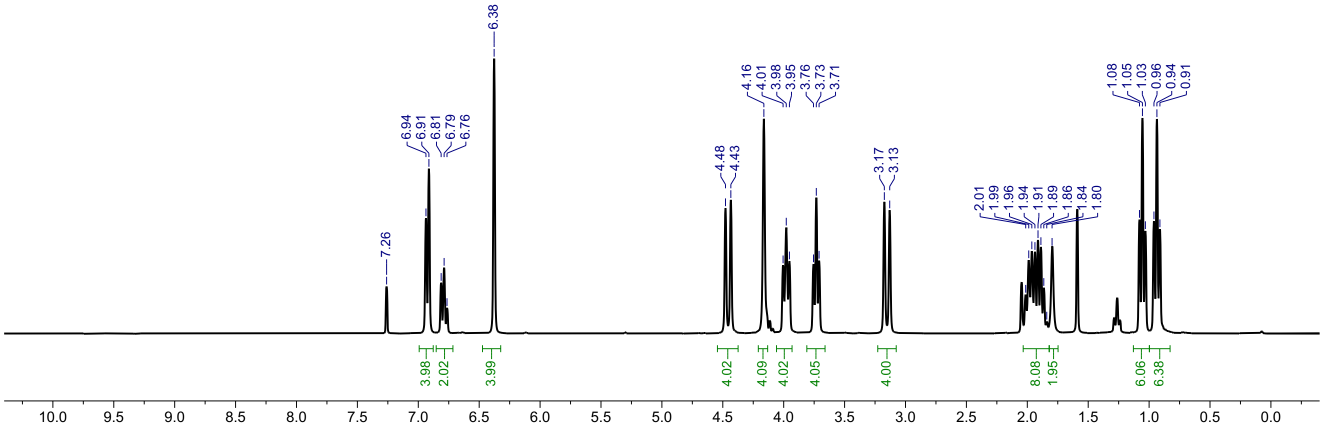

Figure S7. ^{1}H NMR spectrum of **C6** ($CDCl_3$, 300 MHz).

2.7. Preparation of 5,17-bis(chloromethyl)-25,26,27,28-tetrapropoxycalix[4]arene (C7)

Under N_2: to a solution of **C6** (1.31 g, 2.00 mmol) in CH_2Cl_2 (25 mL) cooled to -78 °C was added $SOCl_2$ (0.44 mL, 6.0 mmol) dropwise. The reaction was stirred for 30 mins while warming to ambient temperature and then stirred for a further 2 h. In air: the volatiles were removed *in vacuo* and the crude material washed with ice cold pentane (10 mL). Crystallisation from hot pentane (*ca.* 250 mL) afforded the pure product. Yield: 1.20 g (1.74 mmol, 87%; white solid). Spectroscopic data are consistent with previous reports.[4]

^{1}H NMR ($CDCl_3$, 300 MHz): δ 6.71–6.61 (m, 6H, *m*-Ar+*p*-Ar), 6.58 (s, 4H, *m*-Ar^{Cl}), 4.43 (d, $^2J_{HH}$ = 13.3, 4H, $ArC\underline{H}_2Ar^{Cl}$), 4.24 (s, 4H, CH_2Cl), 3.87 (t, $^3J_{HH}$ = 7.5, 4H, OCH_2), 3.82 (t, $^3J_{HH}$ = 7.5, 4H, OCH_2), 3.14 (d, $^2J_{HH}$ = 13.4, 4H, $ArC\underline{H}_2Ar^{Cl}$), 1.93 (hex, $^3J_{HH}$ = 7.3, 4H, $C\underline{H}_2CH_3$), 1.90 (hex, $^3J_{HH}$ = 7.3, 4H, $C\underline{H}_2CH_3$), 1.00 (t, $^3J_{HH}$ = 7.6, 6H, $CH_2C\underline{H}_3$), 0.97 (t, $^3J_{HH}$ = 7.6, 6H, $CH_2C\underline{H}_3$).

LR ESI-MS (positive ion): 711.5 ($[M+Na]^+$, calcd 711.3) *m*/*z*.

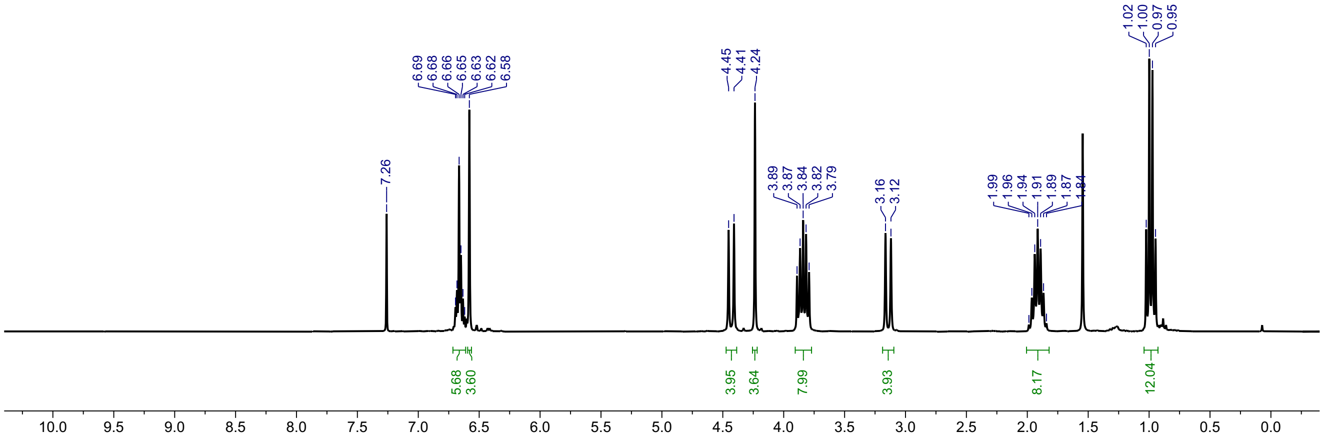

Figure S8. ^1H NMR spectrum of **C7** ($CDCl_3$, 300 MHz).

2.8. Preparation of 5,17-bis(bromomethyl)-25,26,27,28-tetrapropoxycalix[4]arene (C8)

Under N_2: to a solution of **C6** (2.00 g, 3.06 mmol) in CH_2Cl_2 (250 mL) was added PBr_3 (0.316 mL, 63.4 mmol) dropwise at ambient temperature and the reaction stirred for 10 mins. In air: the solution was washed with $NaHCO_{3(aq)}$ (satd., 3×250 mL) and extracted into CH_2Cl_2 (3×50 mL). The organic phase was washed with H_2O (3×250 mL), dried over $MgSO_4$, and dried *in vacuo*. Yield: 2.25 g (2.89 mmol, 93%; white solid). Spectroscopic data are consistent with previous reports.[10]

^{1}H NMR ($CDCl_3$, 300 MHz): δ 6.69–6.61 (m, 6H. *m*-Ar+*p*-Ar), 6.59 (s, 4H, *m*-Ar^{Br}), 4.41 (d, $^2J_{HH}$ = 13.5, 4H, $ArC\underline{H}_2Ar^{Br}$), 4.17 (s, 4H, CH_2Br), 3.85 (t, $^3J_{HH}$ = 7.7, 4H, OCH_2), 3.82 (t, $^3J_{HH}$ = 7.6, 4H, OCH_2), 3.13 (d, $^2J_{HH}$ = 13.4, 4H, $ArC\underline{H}_2Ar^{Br}$), 2.03–1.80 (m, 8H, 2×$C\underline{H}_2CH_3$), 0.99 (t, $^3J_{HH}$ = 7.5, 6H, $CH_2C\underline{H}_3$), 0.97 (t, $^3J_{HH}$ = 7.5, 6H, $CH_2C\underline{H}_3$).

LR ESI-MS (positive ion): 801.3 ([*M*+Na]$^+$, calcd 801.2) *m*/*z*.

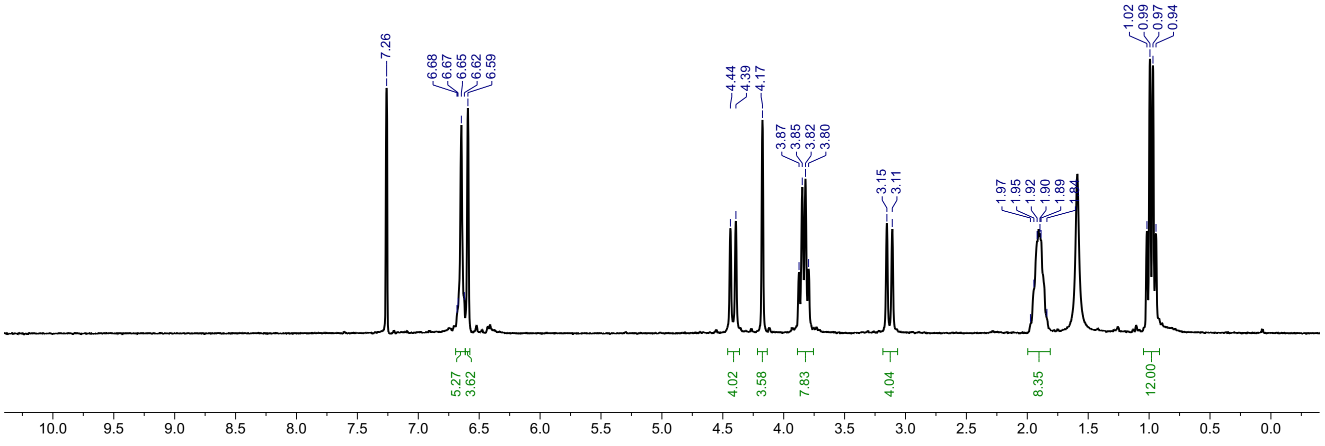

Figure S9. ^1H NMR spectrum of **C8** ($CDCl_3$, 300 MHz).

2.9. Preparation of CxP_2

Method A: To a solution of **C7** (1.0 g, 1.5 mmol) in THF (25 mL) cooled to -78 °C was added a solution of $KPPh_2$ (0.5 M in THF, 8.7 mL, 4.4 mmol) dropwise with stirring. The reaction was warmed to ambient temperate overnight and then refluxed at 70 °C for 2 h. The resulting suspension was washed with $NH_4Cl_{(aq)}$ (satd., 3×30 mL, argon-sparged), H_2O (3×30 mL, argon-sparged), then dried over $MgSO_4$ and filtered. The solution was to *ca*. 5 mL *in vacuo* and the product precipitated by addition of excess hexane (*ca*. 100 mL). Additional material was obtained by concentrating the supernatant to *ca.* 50 mL *in vacuo*. The product was isolated by filtration and the combined batches dried *in vacuo*. Yield: 1.00 g (1.01 mmol, 67%; white crystalline solid).

Method B: To solution of **C8** (0.45 g, 0.58 mmol) in THF (15 mL) cooled to -78 °C was added a solution of $KPPh_2$ (0.5 M in THF, 3.5 mL, 1.75 mmol) dropwise with stirring. The reaction was warmed to ambient temperate overnight, and the resulting suspension washed with $NH_4Cl_{(aq)}$ (satd., 3×20 mL, argon-sparged), H_2O (3×20 mL, argon-sparged), then dried over $MgSO_4$ and filtered. The solution was reduced to *ca*. 5 mL *in vacuo* and the product precipitated by addition of excess hexane (*ca*. 100 mL). Additional material was obtained by concentrating the supernatant to *ca.* 50 mL *in vacuo*. The product was isolated by filtration and the combined batches dried under high vacuum. Yield: 463 mg (46.8 mmol, 81%; white microcrystalline solid).

Spectroscopic data are consistent with previous reports.[4]

^{1}H NMR (CD_2Cl_2, 500 MHz,): *δ* 7.47–7.41 (m, 8H, *o*-Ph), 7.37–7.33 (m, 12H, *m*-Ph+*p*-Ph), 6.75 (s, 4H, *m*-Ar^P), 6.17 (t, $^3J_{HH}$ = 7.5, 2H, *p*-Ar), 5.88 (d, $^3J_{HH}$ = 7.6, 4H, *m*-Ar), 4.31 (d, $^2J_{HH}$ = 13.2, 4H, $ArC\underline{H}_2Ar^P$), 3.89 (t, $^3J_{HH}$ = 6.8, 4H, OCH_2), 3.62 (t, $^3J_{HH}$ = 6.8, 4H, OCH_2), 3.37 (s, 4H, CH_2P), 2.96 (d, $^2J_{HH}$ = 13.3, 4H, $ArC\underline{H}_2Ar^P$), 1.89 (hex, $^3J_{HH}$ = 8.0, 4H, $C\underline{H}_2CH_3$), 1.84 (hex, $^3J_{HH}$ = 7.2, 4H, $C\underline{H}_2CH_3$), 1.05 (t, $^3J_{HH}$ = 7.4, 6H, $CH_2C\underline{H}_3$), 0.88 (t, $^3J_{HH}$ = 7.5, 6H, $CH_2C\underline{H}_3$).
^{31}P{^{1}H} NMR (CD_2Cl_2, 162 MHz): *δ* -10.0 (s).
LR ESI-MS (positive ion): 1097.2 ($[M+Ag]^+$, calcd 1097.4) *m*/*z*.

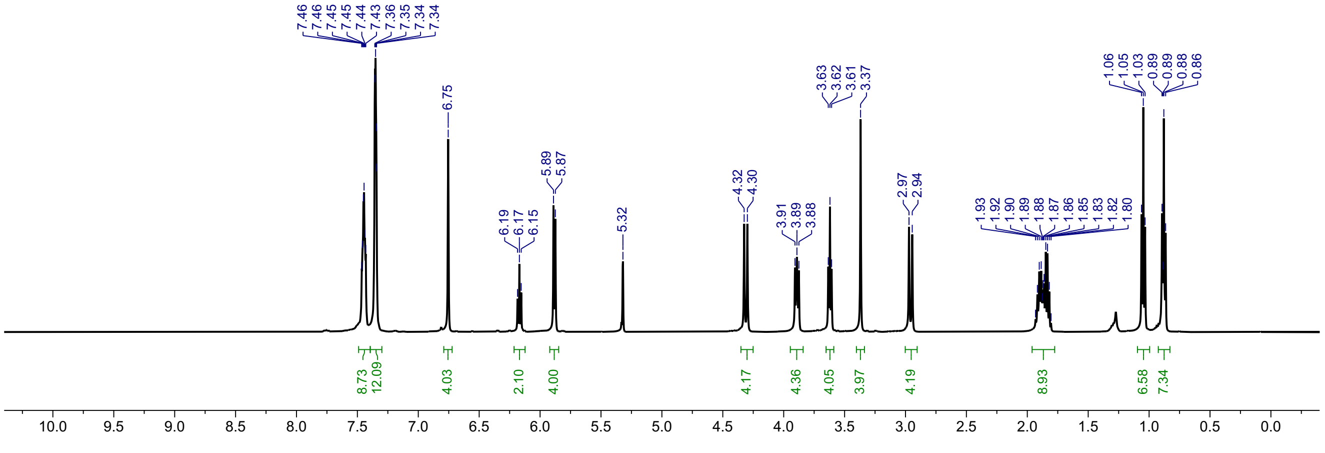

Figure S10. ^{1}H NMR spectrum of CxP_2 (CD_2Cl_2, 500 MHz).

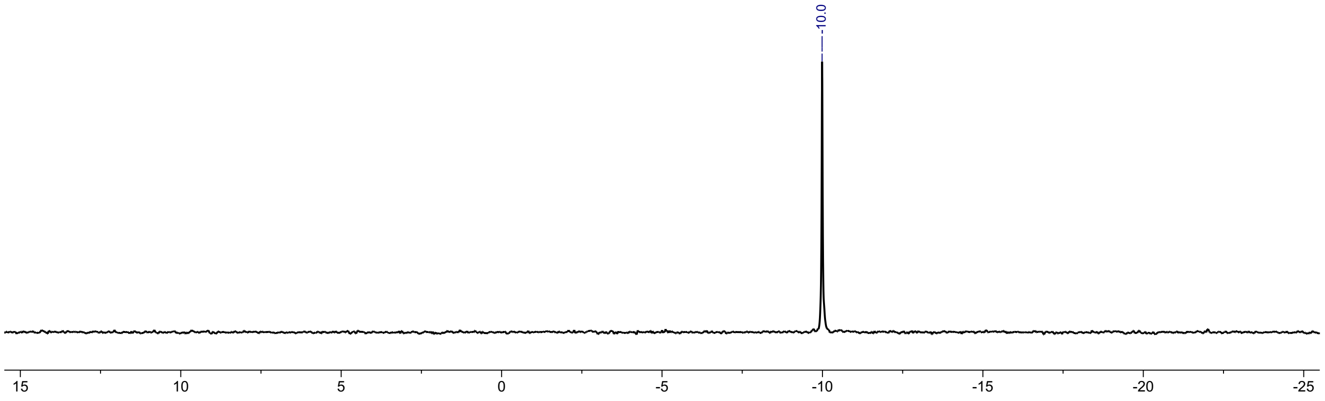

Figure S11. $^{31}P\{^1H\}$ NMR spectrum of CxP_2 (CD_2Cl_2, 162 MHz).

3. Synthesis and reactivity of rhodium triphenylphosphine complexes

3.1. Preparation of *trans*-[Rh(biph)$(PPh_3)_2$(κ^1-$ClCH_2Cl$)][Al$(OR^F)_4$] (2-DCM)

Method A: A suspension of *trans*-[Rh(biph)$(PPh_3)_2$Cl] (326.1 mg, 400.0 μmol) and Li[Al$(OR^F)_4$] (409.1 mg, 420.0 μmol) in anhydrous CH_2Cl_2 (5 mL) over activated 3 Å molecular sieves (*ca*. 100 mg) was stirred at ambient temperature for 18 h. The reaction was filtered, and the product precipitated by addition of excess anhydrous hexane (*ca.* 40 mL), isolated by decantation, and dried in *in vacuo*. Yield: 628.1 mg (359.6 μmol, 96%; orange solid).

Method B: The product can be generated quantitively *in situ* by treatment of **2-OH₂** (17.6 mg, 10.0 μmol) in CD_2Cl_2 (0.5 mL) within a J. Young valve NMR tube with 3 Å molecular sieves (100–2000 wt%, 1–5 h) at ambient temperature.

Spectroscopic data are consistent with previous reports for the $[BAr^F{}_4]^-$ salt.[2]

^{1}H NMR (500 MHz, CD_2Cl_2): δ 7.51 (t, $^3J_{HH}$ = 7.5, 6H, *p*-Ph), 7.33 (t, $^3J_{HH}$ = 7.7, 12H, *m*-Ph), 7.07–6.99(m, 14H, *o*-Ph+6-biph), 6.87 (t, $^3J_{HH}$ = 7.3, 2H, 4-biph), 6.76 (t, $^3J_{HH}$ = 7.7, 2H, 5-biph), 6.65 (d, $^3J_{HH}$ = 7.5, 2H, 3-biph).
$^{13}C\{^1H\}$ NMR (126 MHz, CD_2Cl_2): δ 154.8 (dt, $^1J_{RhC}$ = 39, $^2J_{PC}$= 10, 1-biph), 150.0 (s, 2-biph), 134.0 (vt, J_{PC} = 12, *o*-Ph), 132.4 (s, *p*-Ph), 131.7 (s, 6-biph), 129.6 (vt, J_{PC} = 10, *m*-Ph), 127.0 (vt, J_{PC} = 48, *i*-Ph), 126.5 (s, 5-biph), 125.3 (s, 4-biph), 123.6 (s, 3-biph), 121.8 (q, $^1J_{FC}$ = 294, CF_3).
$^{31}P\{^1H\}$ NMR (162 MHz, CD_2Cl_2): δ 19.7 (d, $^1J_{RhP}$ = 119).
HR ESI-MS (positive ion): 779.1493 ($[M–Cl]^+$, calcd 779.1498) *m*/*z*.

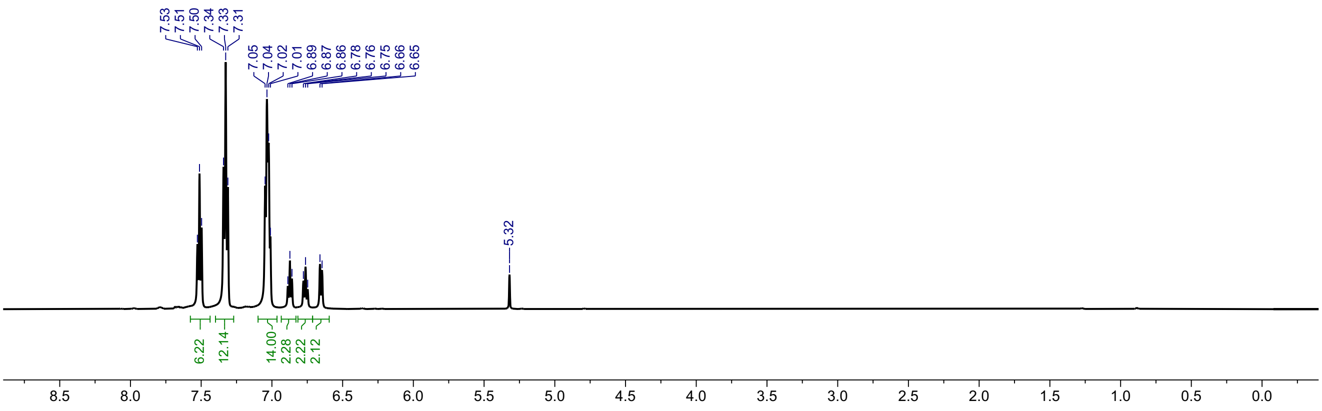

Figure S12. ^{1}H NMR spectrum of **2-DCM** (CD_2Cl_2, 500 MHz).

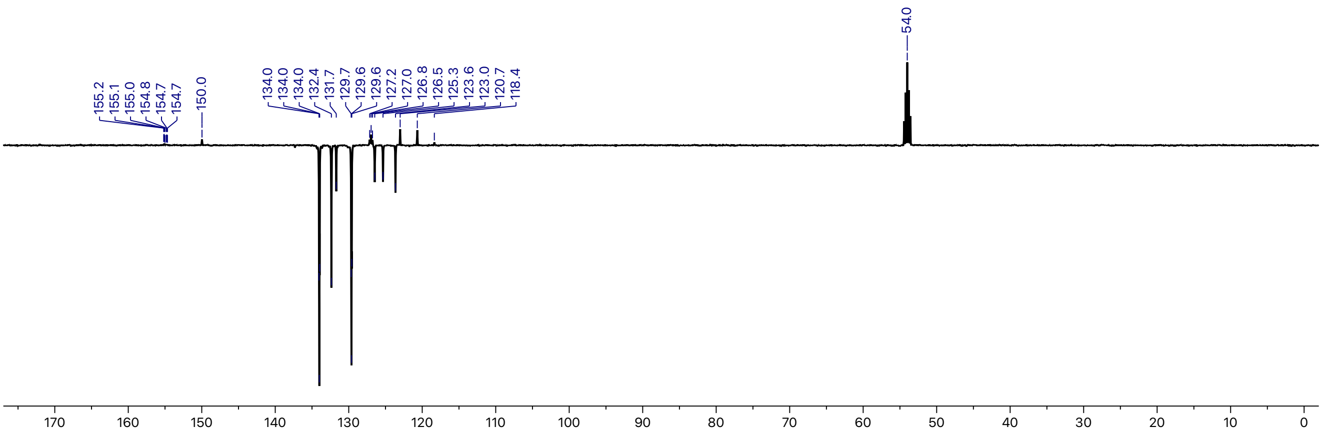

Figure S13. ^{13}C{^{1}H} APT NMR spectrum of **2-DCM** (CD_2Cl_2, 126 MHz).

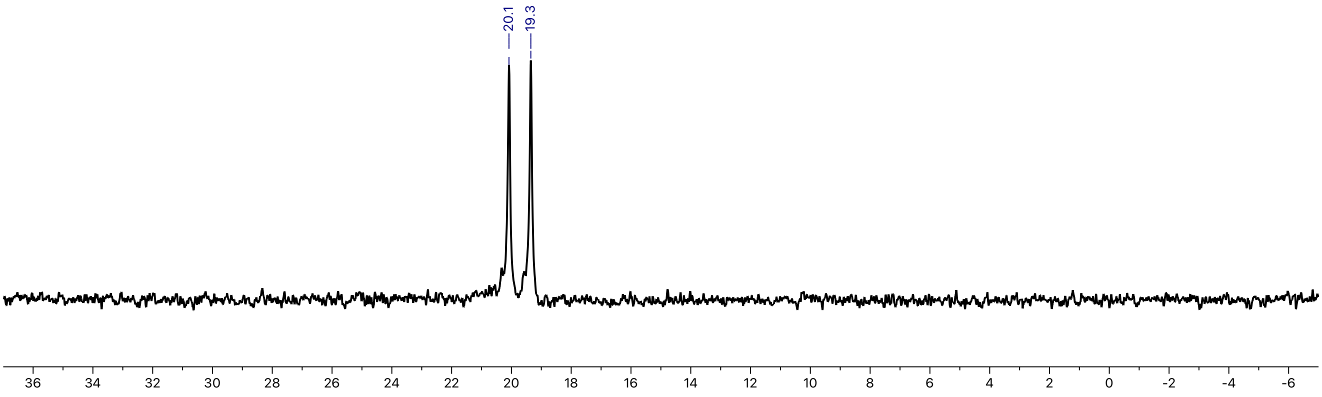

Figure S14. ^{31}P{^{1}H} NMR spectrum of **2-DCM** (CD_2Cl_2, 162 MHz).

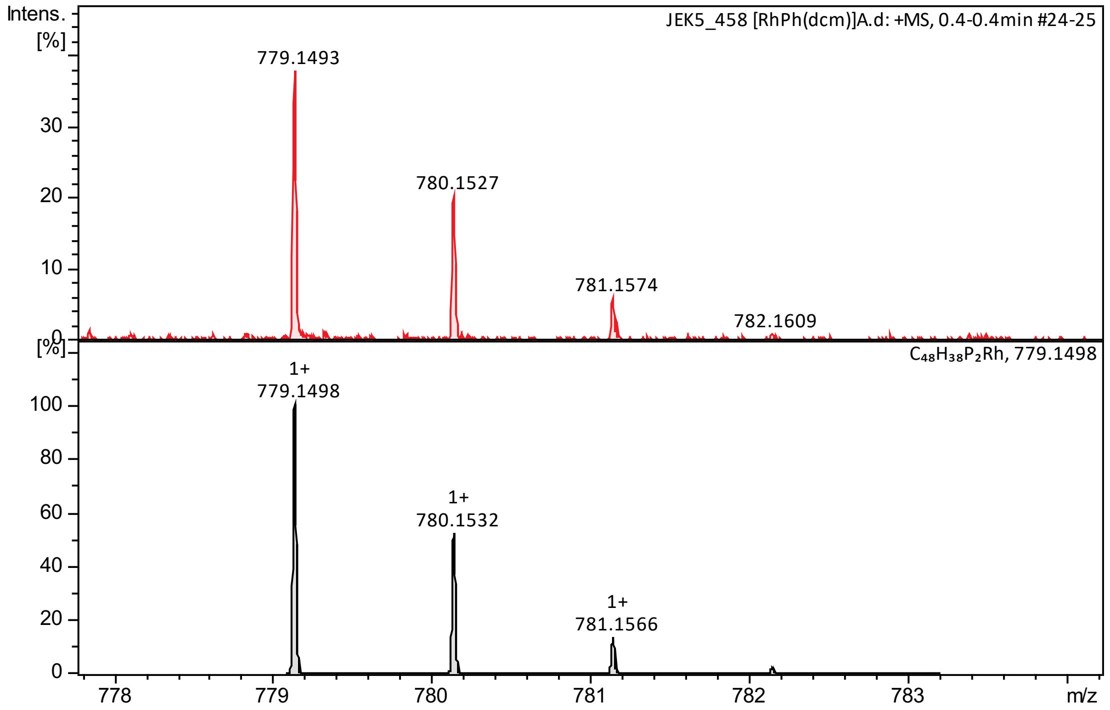

Figure S15. High-resolution ESI mass spectrum of **2-DCM** (top: measured, bottom: calcd.).

3.2. Stability of 2-DCM under dinitrogen.

A solution of **2-DCM** (8.7 mg, 5.00 µmol) in CD_2Cl_2 (0.5 mL) within a J. Young valve NMR tube under an atmosphere of argon (1 atm; passed over activated 3 Å molecular sieves) was freeze-pump-thaw degassed and placed under an atmosphere of dinitrogen (1 atm; passed over activated 3 Å molecular sieves). No meaningful spectroscopic changers were observed after standing at ambient temperature for 18 h.

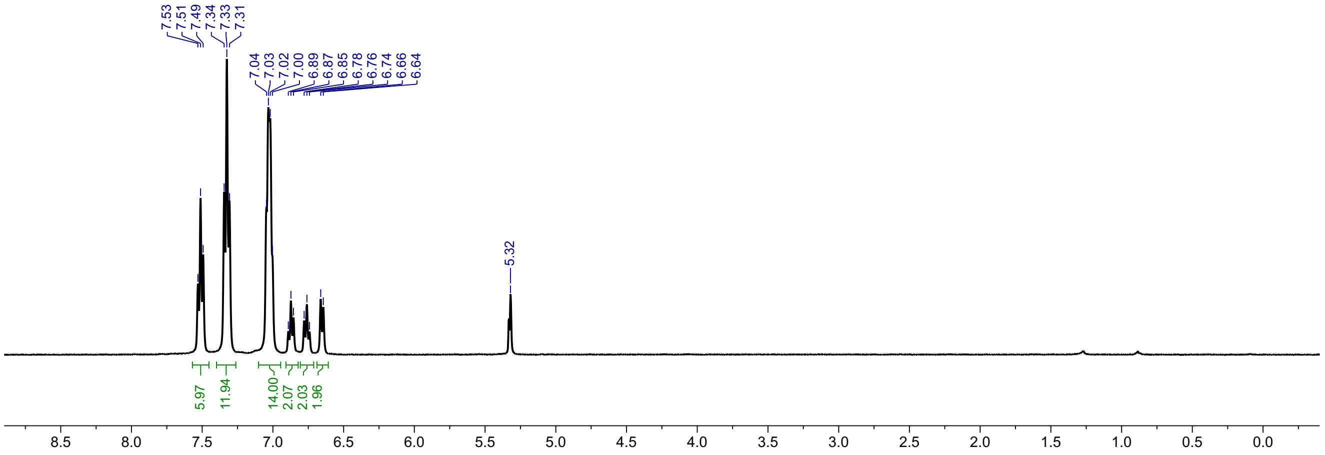

Figure S16. ^{1}H NMR spectrum of **2-DCM** under dinitrogen (CD_2Cl_2, 400 MHz).

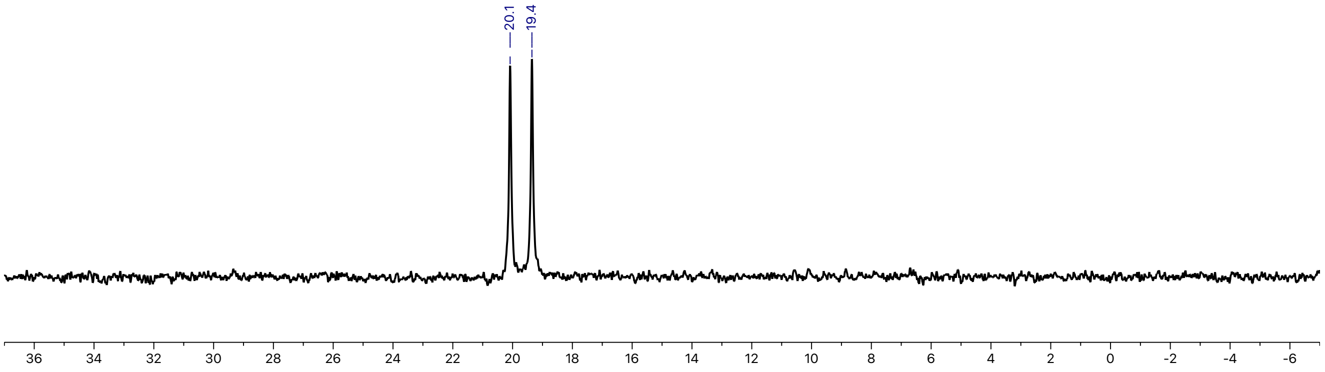

Figure S17. $^{31}P\{^1H\}$ NMR spectrum of **2-DCM** under dinitrogen (CD_2Cl_2, 162 MHz).

3.3. Preparation of *trans*-[Rh(biph)(PPh_3)$_2$(OH_2)][Al(OR^F)$_4$] (2-OH$_2$)

Method A: A suspension of *trans*-[Rh(biph)(PPh_3)$_2$Cl] (360 mg, 442 μmol) and Li[Al(OR^F)$_4$] (475 mg, 486 μmol) in CH_2Cl_2 (5 mL) was stirred at ambient temperature for 18 h. The reaction was filtered and H_2O (7.0 μL, 4.4 mmol) was added to the supernatant. The crude product was precipitated by addition of excess hexane (*ca*. 40 mL) and isolated by filtration. The crude product was extracted into CH_2Cl_2 (*ca.* 10 mL), filtered, and the solvent removed *in vacuo* to afford the product. Yield: 725.5 mg (411.1 μmol, 93%; orange solid).

Method B: To a solution of **2-DCM** (200 mg, 112 μmol) in CH_2Cl_2 (1 mL) was added excess of H_2O (*ca.* 0.1 mL) at ambient temperature. The solution was agitated briefly, and the product precipitated by addition of excess hexane (*ca.* 20 mL), isolated by filtration, and dried *in vacuo*. Yield: 189.6 mg (107.4 μmol, 96%; orange solid).

Whilst not systematically prepared, the crystal structure of the $[BAr^F{}_4]^-$ salt has previously been reported.[2]

^{1}H NMR (CD_2Cl_2, 500 MHz): δ 7.52 (t, $^3J_{HH}$ = 7.4, 6H, *p*-Ph), 7.35 (t, $^3J_{HH}$ = 7.5, 12H, *m*-Ph), 6.98 (app. q, J = 5.7, 12H, *o*-Ph), 6.93 (d, $^3J_{HH}$ = 7.7, 2H, 6-biph), 6.91 (t, $^3J_{HH}$ = 7.5, 2H, 4-biph), 6.83 (d, $^3J_{HH}$ = 7.3, 2H, 3-biph), 6.74 (t, $^3J_{HH}$ = 7.5, 2H, 5-biph), 2.44 (s, 2H, OH_2).
^{13}C{^{1}H} NMR (CD_2Cl_2, 126 MHz): δ 154.0 (dt, $^1J_{RhC}$ = 39, $^2J_{PC}$ = 9, 1-biph), 150.9 (s, 2-biph), 133.8 (vt, J_{PC} = 12, *o*-Ph), 132.4 (s, *p*-Ph), 132.0 (s, 6-biph), 129.9 (vt, J_{PC} = 10, *m*-Ph), 126.4 (vt, J_{PC} = 47, *i*-Ph), 126.1 (s, 5-biph), 125.0 (s, 4-biph), 123.2 (s, 3-biph), 121.8 (q, $^1J_{FC}$ = 294, CF_3).
^{31}P{^{1}H} NMR (CD_2Cl_2, 162 MHz): δ 22.2 (d, $^1J_{RhP}$ = 119).
HR ESI-MS (positive ion): 779.1492 ($[M\text{–}OH_2]^+$, calcd 779.1498) *m*/*z*.
Anal. Calcd for $C_{64}H_{40}AlF_{36}O_5P_2Rh$ (1764.80 g·mol^{-1}): C, 43.56; H, 2.28; N, 0.00. Found: C, 43.65; H, 2.44; N, 0.00.

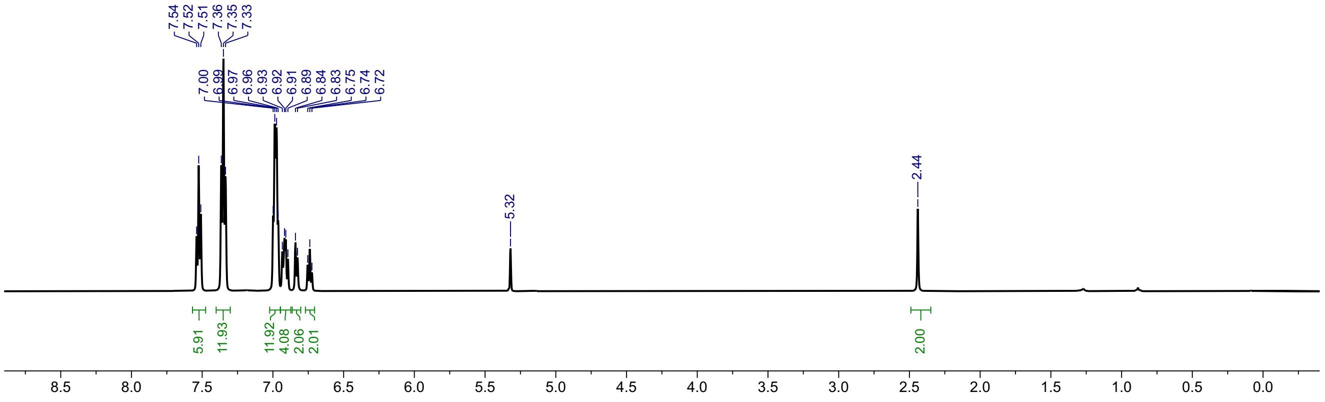

Figure S18. ^{1}H NMR spectrum of **2-OH$_2$** (CD_2Cl_2, 500 MHz).

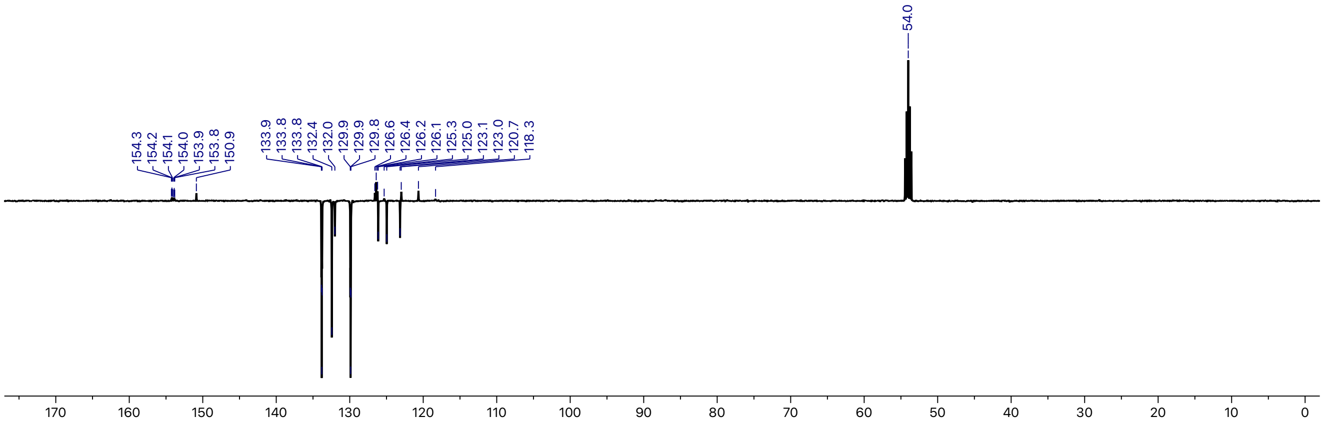

Figure S19. $^{13}C\{^{1}H\}$ APT NMR spectrum of **2-OH$_2$** (CD_2Cl_2, 126 MHz).

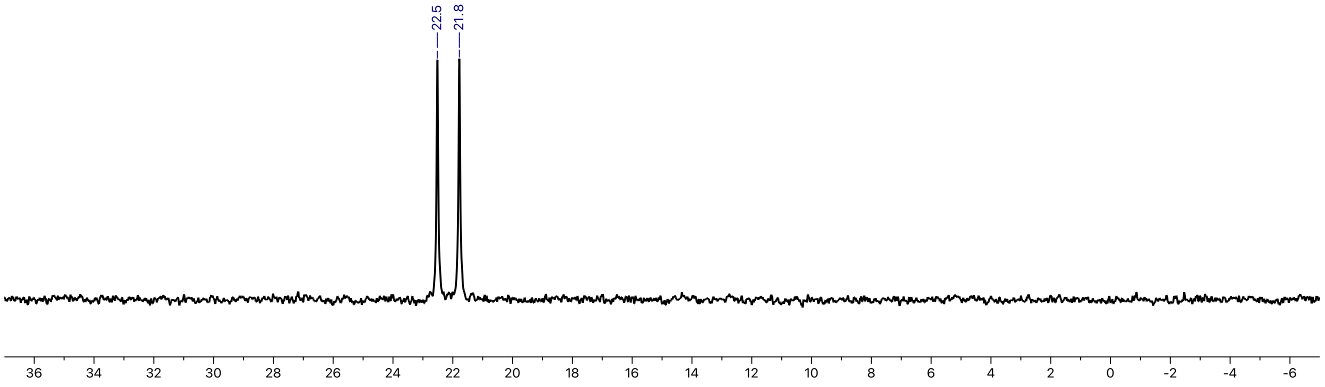

Figure S20. $^{31}P\{^{1}H\}$ NMR spectrum of **2-OH$_2$** (CD_2Cl_2, 162 MHz).

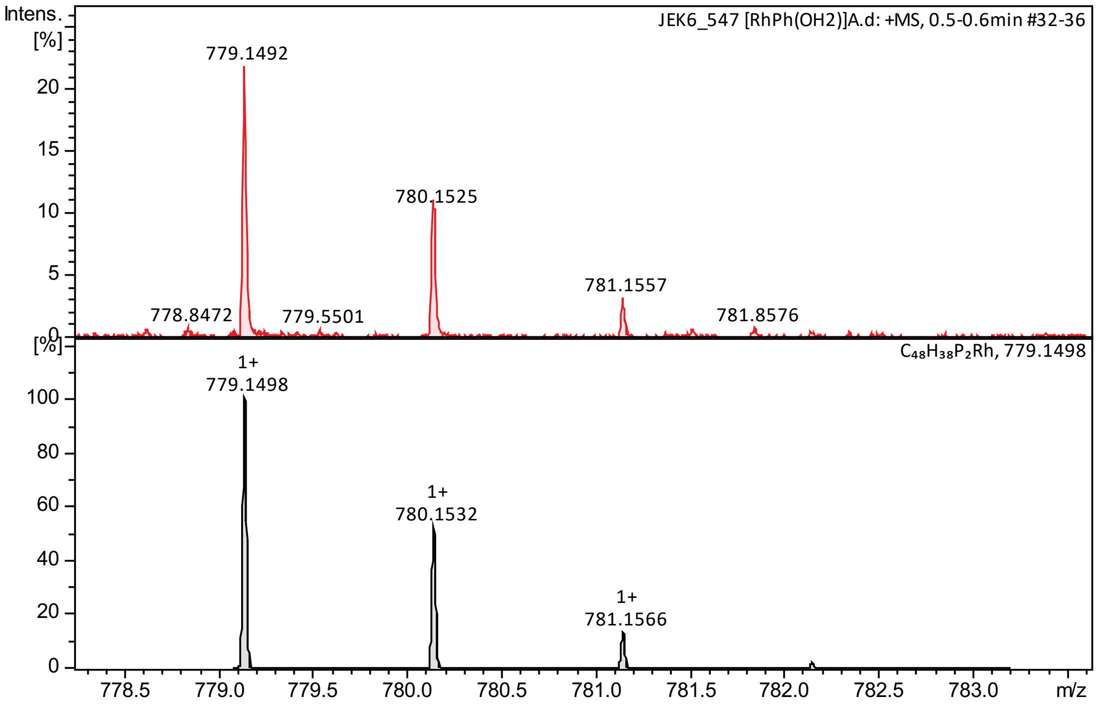

Figure S21. High-resolution ESI mass spectrum of **2-OH$_2$** (top: measured, bottom: calcd).

4. Synthesis and reactivity of rhodium CxP$_2$ complexes

4.1. Preparation of [Rh(biph)(CxP$_2$)(OH$_2$)][Al(OR^F)$_4$] (1-OH$_2$)

A solution of **2-OH$_2$** (176.5 mg, 100.0 µmol) and **CxP$_2$** (100.0 mg, 110.0 µmol) in THF (10 mL) was stirred at ambient temperature for 4 h. H_2O (18 µL, 1 mmol) was added and the reaction stirred for a further 18 h. The solution was then added into an excess of rapidly stirred hexane, precipitating a yellow material which was isolated by filtration and washed with hexane. The separated solid was extracted into CH_2Cl_2 (*ca*. 5 mL) and additional H_2O added (*ca*. 0.1 mL). Slow diffusion of excess hexane (*ca*. 40 mL) into this solution afforded orange crystalline material and a red oily residue. The solid material was isolated by filtration, dried under high vacuum and then, in air, dissolved in *wet* CH_2Cl_2 and passed through a short plug of Al_2O_3 (100% CH_2Cl_2). The solvent was removed *in vacuo* to afford the product. Yield: 97.0 mg (43.5 µmol, 44%; yellow solid). Single crystals were obtained from crystallisation from CH_2Cl_2/hexane at room temperature.

^{1}H NMR (CD_2Cl_2, 500 MHz): δ 7.6–6.0 (m, 34H, Ar$^{H/P}$+Ph+biph), 4.48 (d, $^2J_{HH}$ = 12.7, 4H, ArC$\underline{H}_2$Ar^P), 4.23–4.00 (m, 4H, OCH$_2$), 3.63 (t, $^3J_{HH}$ = 7.6, 4H, OCH$_2$), 3.27 (br, 4H, C$\underline{H}_2$P), 3.09 (br d, $^2J_{HH}$ = 12.7, 4H, ArC$\underline{H}_2$Ar^P), 2.16–2.08 (m, 4H, C$\underline{H}_2$CH$_3$), 1.89 (hex, $^3J_{HH}$ = 7.4, 4H, C$\underline{H}_2$CH$_3$), 1.03 (t, $^3J_{HH}$ = 7.4, 6H, CH$_2$C$\underline{H}_3$), 0.95 (t, $^3J_{HH}$ = 7.5, 6H, CH$_2$C$\underline{H}_3$), 0.84 (s, 2H, OH$_2$).

^{13}C{^{1}H} NMR (CD_2Cl_2, 126 MHz, selected data): δ 157.5 (s, *i*-Ar$^{H/P}$), 156.1 (s, *i*-Ar$^{H/P}$), 136.6 (s, *o*-Ar$^{H/P}$), 136.4 (br, *o*-Ar$^{H/P}$),128.6 (s, *p*-Ar^P), 121.8 (q, $^1J_{FC}$ = 294, CF$_3$). 78.9 (s, OCH$_2$), 77.2 (s, OCH$_2$), 32.0 (vt, J_{PC} = 25, CH$_2$P), 31.5 (s, Ar$\underline{C}$H$_2$Ar^P), 23.9 (s, $\underline{C}$H$_2$CH$_3$), 23.5 (s, $\underline{C}$H$_2$CH$_3$), 10.9 (s, CH$_2\underline{C}$H$_3$), 10.0 (s, CH$_2\underline{C}$H$_3$).

^{I31}P{^{1}H} NMR (CD_2Cl_2, 162 MHz): δ 13.2 (d, $^1J_{RhP}$ = 120).

^{31}P{^{1}H} NMR (C_6H_5F, 162 MHz,): δ 13.0 (d, $^1J_{RhP}$ = 120).

^{19}F{^{1}H} NMR (CD_2Cl_2, 377 MHz): δ -75.75 (s).

FT-IR (ATR): *ν*(O-H) 3528, 3457 cm^{-1}

HR ESI-MS (positive ion): 1261.4545 ([*M*]$^+$, calcd 1261.4531) *m*/*z*.

Anal. Calcd for $C_{94}H_{80}AlF_{36}O_9P_2Rh$ (2229.44 g·mol^{-1}): C, 50.64; H, 3.62; N, 0.00. Found: C, 50.53; H, 3.58; N, 0.00.

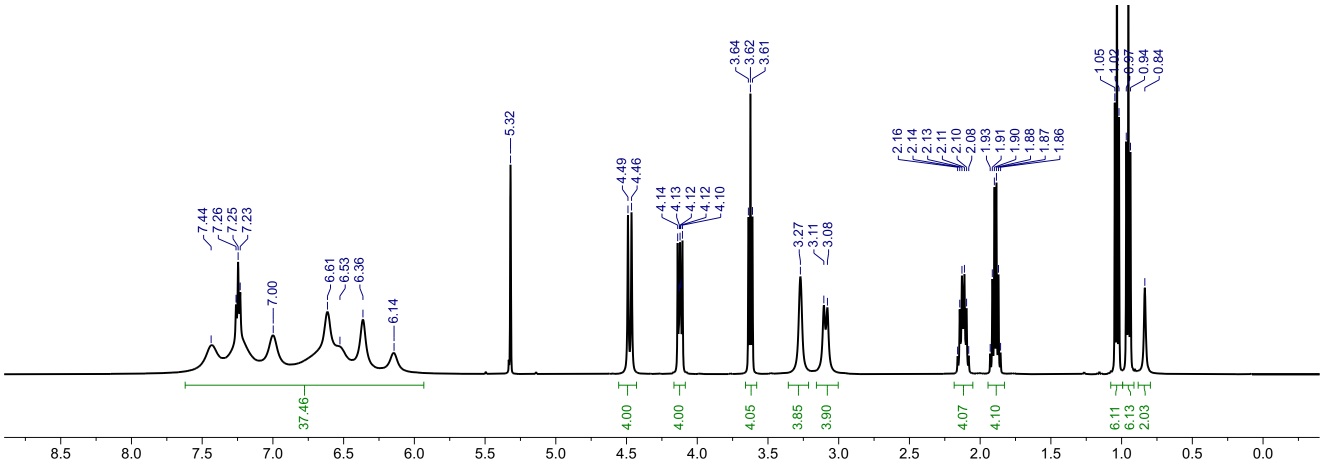

Figure S22. ^{1}H NMR spectrum of **1-OH₂** (CD_2Cl_2, 500 MHz).

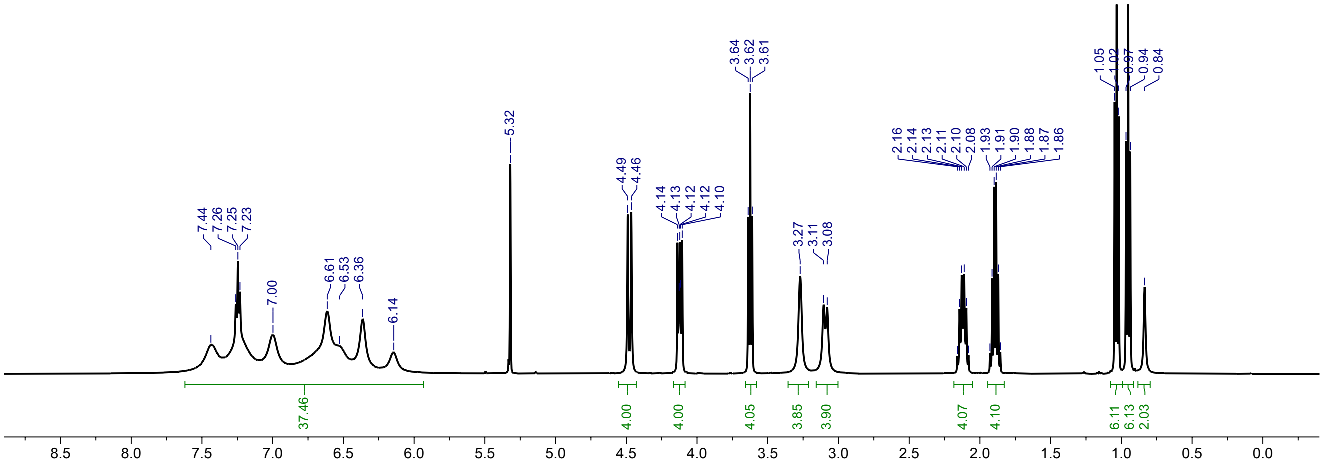

Figure S23. $^{13}C\{^1H\}$ APT NMR spectrum of **1-OH₂** (CD_2Cl_2, 126 MHz).

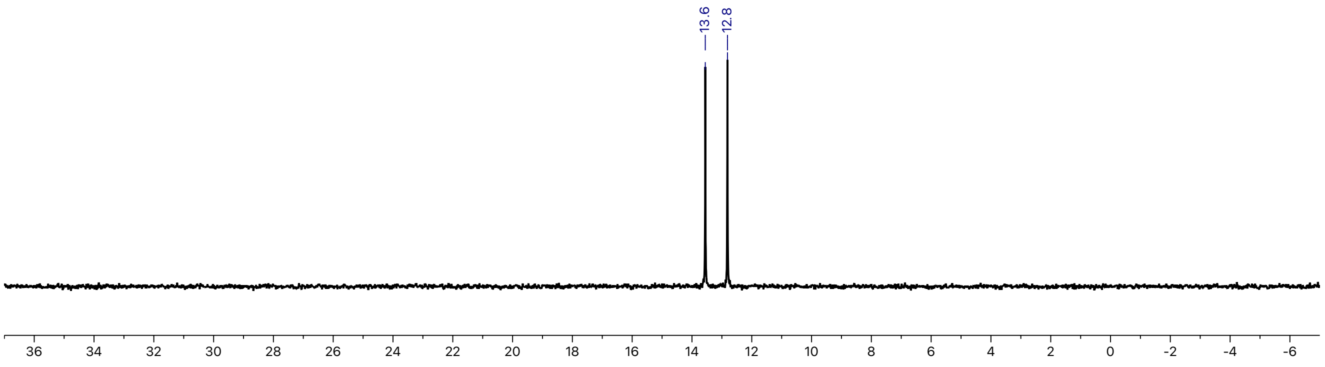

Figure S24. $^{31}P\{^1H\}$ NMR spectrum of **1-OH₂** (CD_2Cl_2, 162MHz).

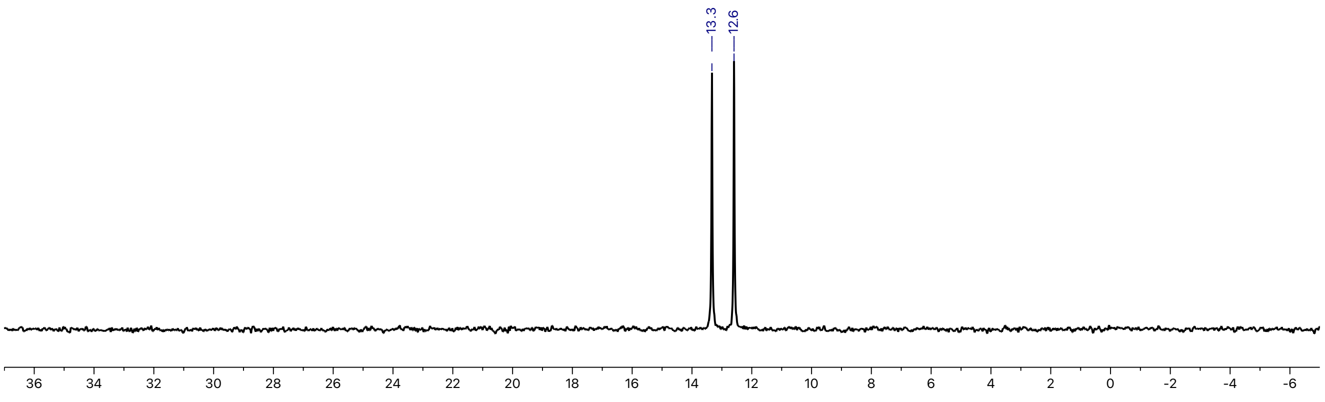

Figure S25. $^{31}P\{^1H\}$ NMR spectrum of **1-OH₂** (C_6H_5F, 162 MHz).

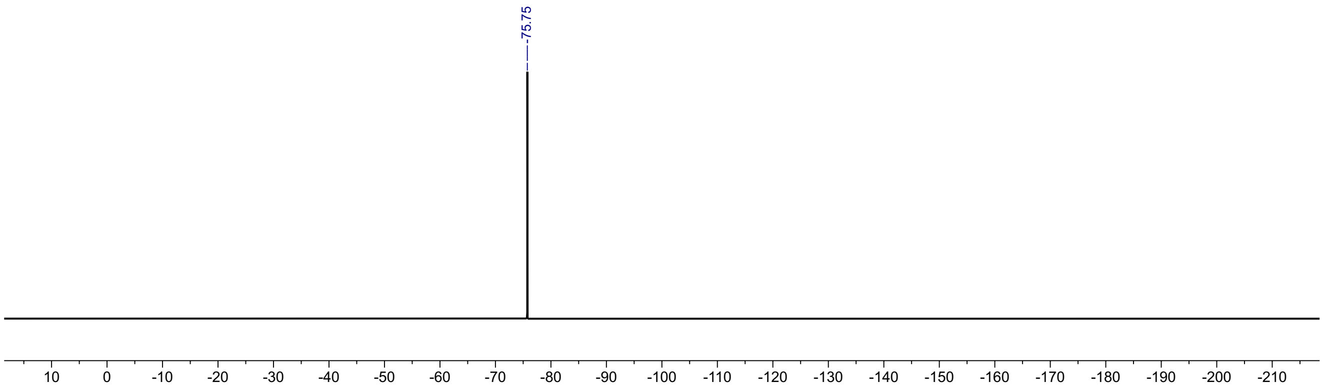

Figure S26. $^{19}F\{^1H\}$ NMR spectrum of **1-OH₂** (CD_2Cl_2, 377 MHz).

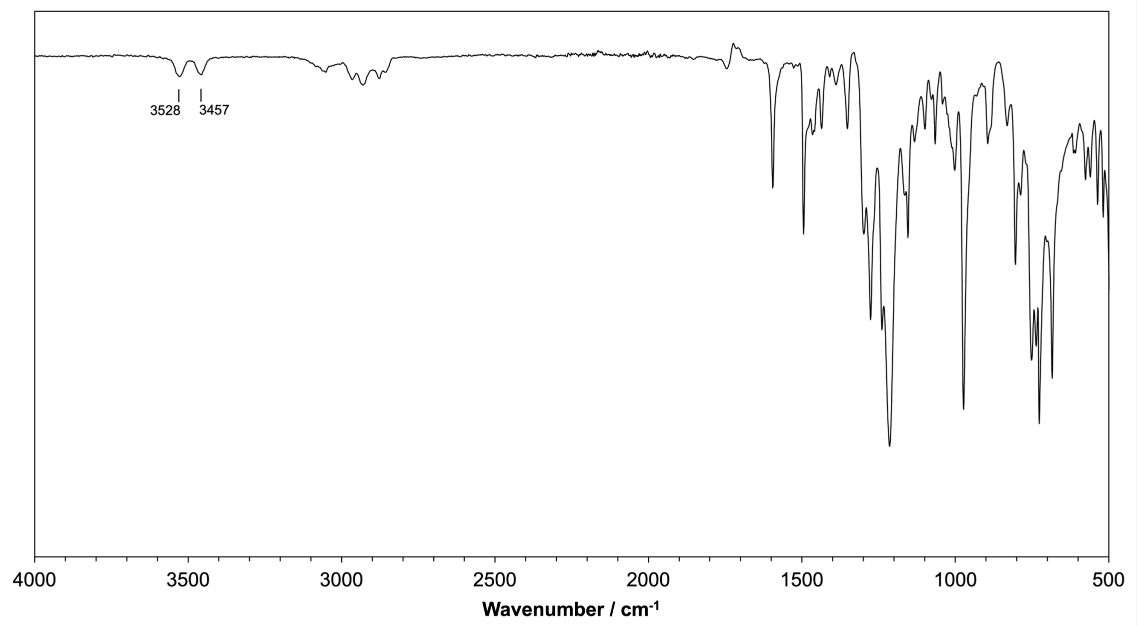

Figure S27. ATR-IR spectrum of **1-OH₂**.

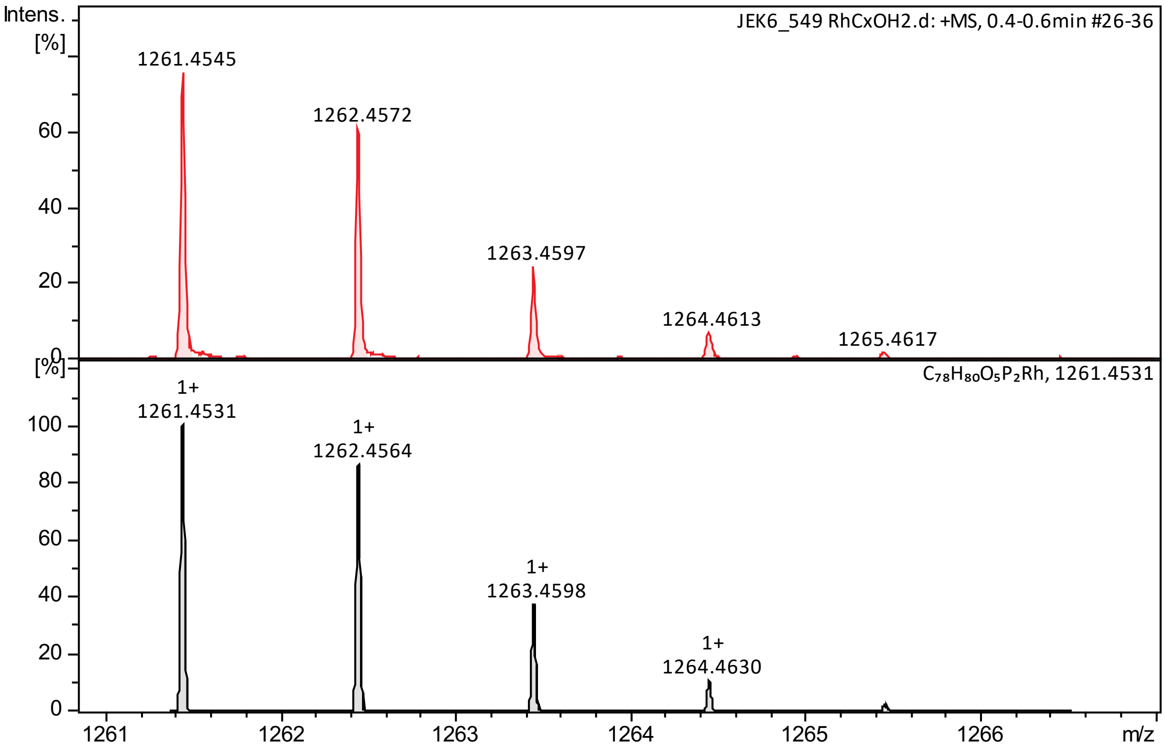

Figure S28. High-resolution ESI mass spectrum of **1-OH$_2$** (top: measured, bottom: calcd.)

4.2. Reaction of 1-OH$_2$ with D$_2$O

To a solution of **1-OH$_2$** (5.0 mg, 2.24 µmol) in CD_2Cl_2 (0.5 mL) prepared within a J. Young valve NMR tube was added D_2O (4.5 µL, 224 µmol). The tube was mounted onto a rotating disc and inverted at *ca.* 0.5 Hz at room temperature. After 48 h, the ^{1}H NMR spectrum indicated formation of **1-OD$_2$** with concomitant liberation of H_2O (δ 1.53).

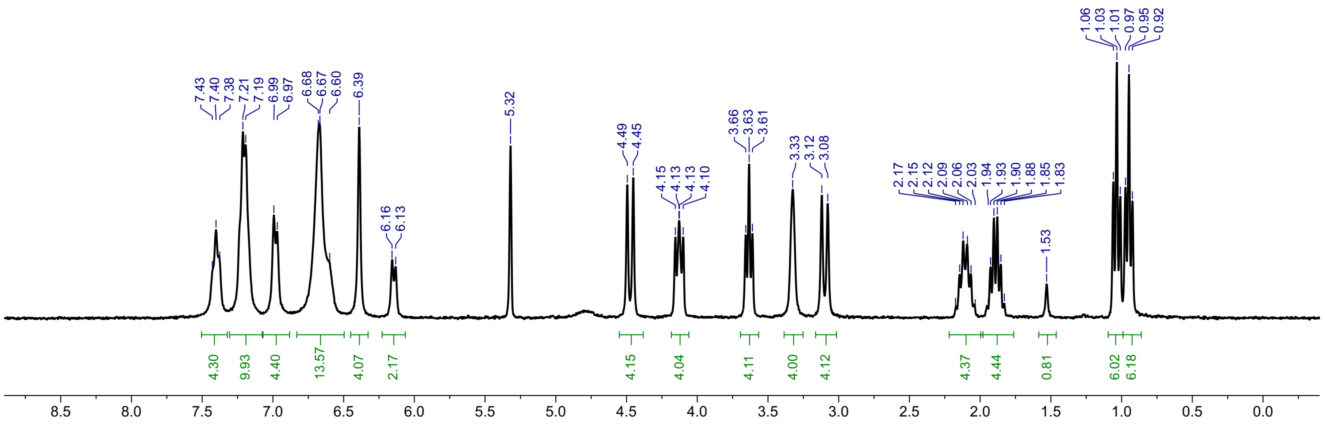

Figure S29. ^{1}H NMR spectrum recorded after treatment of **1-OH$_2$** with D_2O (CD_2Cl_2, 300 MHz).

4.3. Dehydration of 1-OH₂ using [ZrCp₂Me₂] in dichloromethane under dinitrogen

A solution of **1-OH$_2$** (11.1 mg, 5.00 μmol) and [ZrCp$_2$Me$_2$] (6.2 mg, 25.0 μmol; δ_{1H} -0.39, 6.12) in CD_2Cl_2 (0.5 mL) prepared within a J. Young valve NMR under an atmosphere of argon (1 atm; passed over activated 3 Å molecular sieves). The solution was freeze-pump-thaw degassed and placed under an atmosphere of dinitrogen (1 atm; passed over activated 3 Å molecular sieves). The ensuring reaction was periodically monitored by NMR spectroscopy at 298 K, which indicated smooth conversion of **1-OH$_2$** into a 6:5 mixture of **1-N$_2$** (δ_{31P} 16.1) and **1-DCM** (δ_{31P} 4.4) with concomitant formation of [ZrCp$_2$Me]$_2$O (δ_{1H} 5.99, 0.00) and methane (δ_{1H} 0.23) within 24 h. On one occasion a single crystal of **1-N$_2$** was obtained from slow diffusion of hexane into the reaction mixture at room temperature. Repeating the reaction under argon in CH_2Cl_2 resulted exclusively in formation of **1-DCM**.

Data for **1-N$_2$**:

^{1}H NMR (CD_2Cl_2, 400 MHz, selected data): δ 4.49 (d, $^2J_{HH}$ =12.7, 4H, ArCH$_2$Ar^P), 4.14–4.06 (m, 4H, OCH$_2$), 3.65 (t, $^3J_{HH}$ = 7.2, 4H, OCH$_2$), 3.15 (vbr, 8H, CH$_2$P+ArCH$_2$Ar^P), 2.17 (hex, $^3J_{HH}$ = 7.4, 4H, C$\underline{H}_2$CH$_3$), 1.91 (hex, $^3J_{HH}$ = 7.3, 4H, C$\underline{H}_2$CH$_3$), 1.05 (t, $^3J_{HH}$ = 7.4, 6H, CH$_2$C$\underline{H}_3$), 0.98 (t, $^3J_{HH}$ = 7.5, 6H, CH$_2$C$\underline{H}_3$).

^{31}P{^{1}H} NMR (CD_2Cl_2, 162 MHz,): δ 16.1 (d, $^1J_{RhP}$ = 117).

Data for **1-DCM**:

^{1}H NMR (CD_2Cl_2, 400 MHz, selected data): δ 4.51 (d, $^2J_{HH}$ = 12.8, 4H, ArCH$_2$Ar^P), 4.26–4.18 (m, 4H, OCH$_2$), 3.85 (br, 4H, CH$_2$P), 3.71 (t, $^3J_{HH}$ = 7.3, 4H, OCH$_2$), 3.17 (d, $^2J_{HH}$ = 13.0, 4H, ArCH$_2$Ar^P), 2.03 (hex, $^3J_{HH}$ = 7.5, 4H, C$\underline{H}_2$CH$_3$), 1.91 (hex, $^3J_{HH}$ = 7.3, 4H, C$\underline{H}_2$CH$_3$), 1.05 (t, $^3J_{HH}$ = 7.4, 6H, CH$_2$C$\underline{H}_3$), 0.95 (t, $^3J_{HH}$ = 7.5, 6H, CH$_2$C$\underline{H}_3$).

^{31}P{^{1}H} NMR (CD_2Cl_2, 162 MHz,): δ 4.4 (d, $^1J_{RhP}$ = 117).

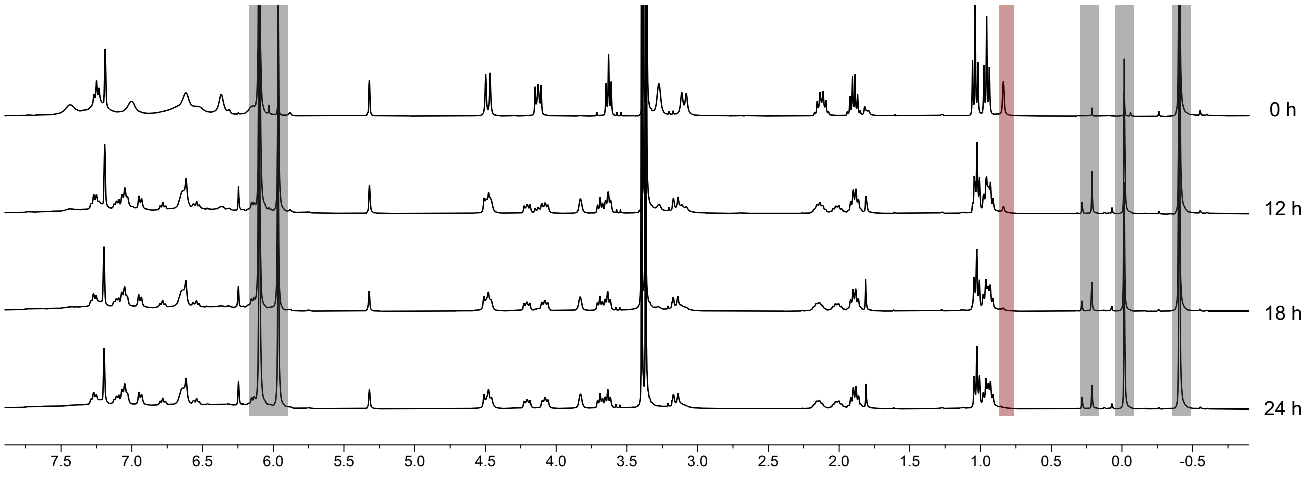

Figure S30. ^{1}H NMR spectra collected during the reaction of **1-OH$_2$** with [ZrCp$_2$Me$_2$] in CD_2Cl_2 under N_2 (400 MHz). Sample contains an internal sealed O=P(OMe)$_3$ reference (δ_{1H} 3.38, $^3J_{PH}$ = 11 Hz); resonances associated with [ZrCp$_2$Me$_2$], [ZrCp$_2$Me]$_2$O, and MeH in grey; coordinated water in **1-OH$_2$** red.

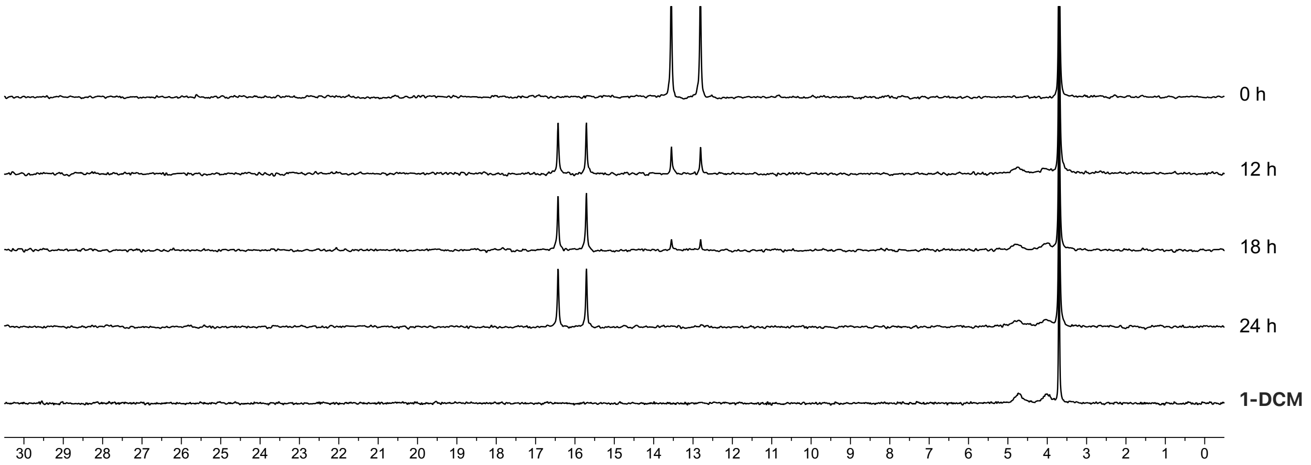

Figure S31. $^{31}P\{^1H\}$ NMR spectra collected during the reaction of **1-OH$_2$** with [$ZrCp_2Me_2$] in CD_2Cl_2 under N_2 and independently synthesised **1-DCM** under argon (162 MHz). Samples contain an internal sealed O=P(OMe)$_3$ reference (δ_{31P} 3.7).

4.4. Dehydration of 1-OH$_2$ using [$ZrCp_2Me_2$] in fluorobenzene under dinitrogen

A solution of **1-OH$_2$** (11.1 mg, 5.00 μmol) and [$ZrCp_2Me_2$] (6.2 mg, 25.0 μmol; δ_{1H} -0.22, 5.87) in C_6H_5F (0.5 mL) prepared within a J. Young valve NMR tube under dinitrogen (1 atm; passed over activated 3 Å molecular sieves). The ensuring reaction was periodically monitored by NMR spectroscopy at 298 K, which indicated smooth conversion of **1-OH$_2$** into **1-N$_2$** (δ_{31P} 16.0) with concomitant formation of [$ZrCp_2Me$]$_2$O (δ_{1H} 5.86, 0.17) and methane (δ_{1H} 0.14) within 13 h. Complete decomposition to an intractable precipitate via a transient intermediate assumed to be 'naked' **1** (δ_{31P} 17.1, $^1J_{RhP}$ = 122 Hz) was observed on a similar timescale when the reaction was repeated under an atmosphere of argon (1 atm, passed over activated 3 Å molecular sieves).

Data for **1-N$_2$**:

^{1}H NMR (CD_2Cl_2, 600 MHz, selected data): δ 4.45 (br, 4H, ArCH$_2$Ar^P), 4.03 (br, 4H, OCH$_2$), 3.52 (br, 4H, OCH$_2$), 3.08 (vbr, 8H, CH$_2$P ArCH$_2$Ar^P), 2.14 (br, 4H, C$\underline{H}_2$CH$_3$), 1.79 (br, 4H, C$\underline{H}_2$CH$_3$), 0.90 (br, 12H, CH$_2$C$\underline{H}_3$+CH$_2$C$\underline{H}_3$).

$^{31}P\{^1H\}$ NMR (C_6H_5F, 243 MHz,): δ 16.0 (d, $^1J_{RhP}$ = 117).

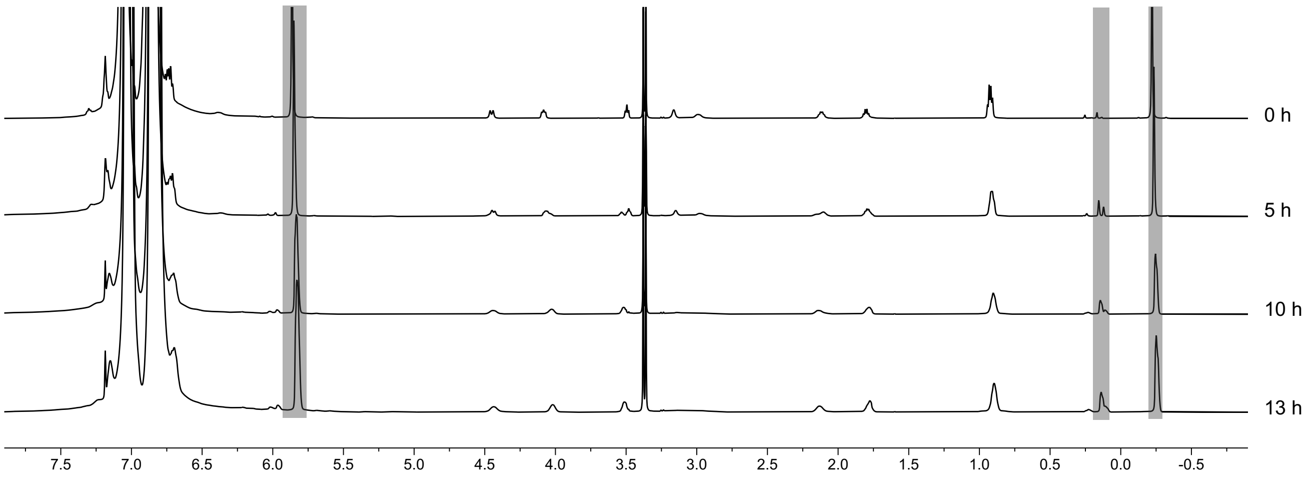

Figure S32. ^{1}H NMR spectra collected during the reaction of **1-OH$_2$** with [ZrCp$_2$Me$_2$] in C_6H_5F under N_2 (600 MHz). Sample contains an internal sealed O=P(OMe)$_3$ reference (δ_{1H} 3.38, $^{3}J_{PH}$ = 11 Hz); resonances associated with [ZrCp$_2$Me$_2$], [ZrCp$_2$Me]$_2$O, and MeH in grey (coordinated water in **1-OH$_2$** is obscured by a methyl resonance).

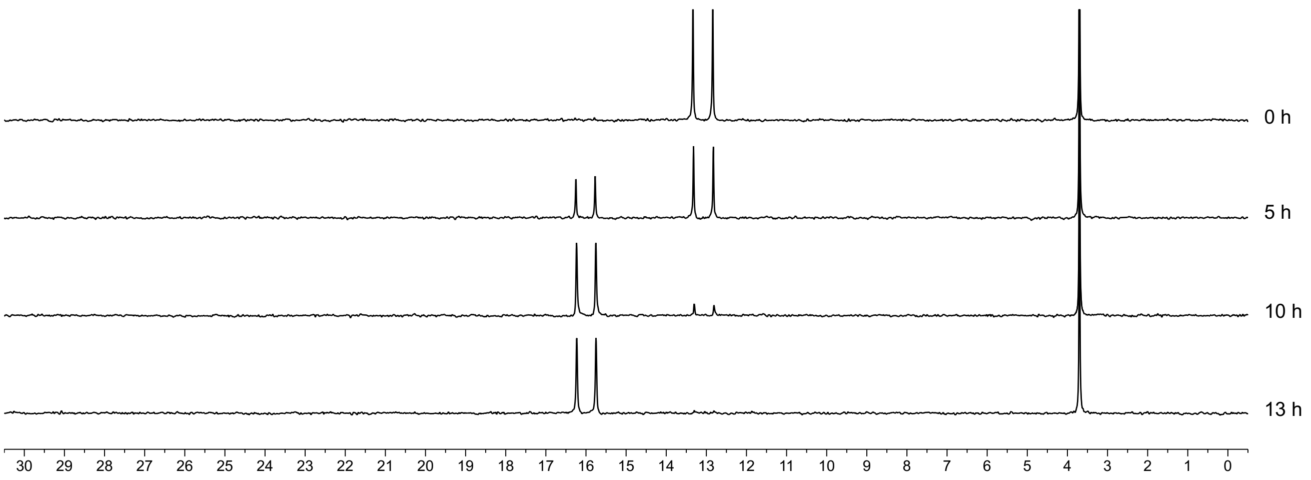

Figure S33. $^{31}P\{^{1}H\}$ NMR spectra collected during the reaction of **1-OH$_2$** with [ZrCp$_2$Me$_2$] in C_6H_5F under N_2 (243 MHz). Sample contains an internal sealed O=P(OMe)$_3$ reference (δ_{31P} 3.7).

4.5. Analysis of 1-N$_2$ by ^{15}N NMR spectroscopy

A 10 mM solution of **1-^{15}N$_2$** was prepared in fluorobenzene (0.5 mL) within a J. Young valve NMR tube under $^{15}N_2$ (1 atm). Only free dinitrogen was observed by ^{15}N NMR spectroscopy.[12]

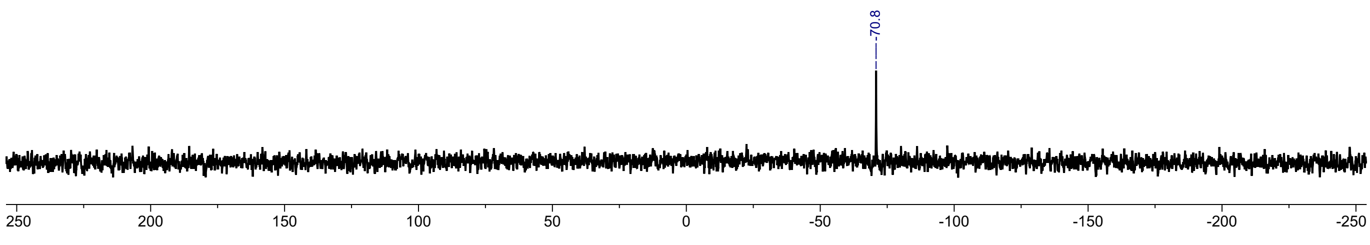

Figure S34. ^{15}N NMR spectra of sample of **1-^{15}N$_2$** in C_6H_5F under $^{15}N_2$ (41 MHz, external CH_3NO_2 ref.)

4.6. Analysis of 1-N_2 by IR spectroscopy

A solution of **1-OH$_2$** (11.1 mg, 5.0 µmol) and [$ZrCp_2Me_2$] (3.1 mg, 12.5 µmol) in C_6H_5F (0.5 mL) was prepared within a J. Young valve NMR tube under dinitrogen (1 atm; passed over activated 3 Å molecular sieves) as described above, monitoring by NMR spectroscopy until full conversion of **1-OH$_2$** into **1-N$_2$** was observed. In a dinitrogen glovebox (<0.1 ppm H_2O and O_2), an aliquot of the sample was transferred into a pre-dried KBr transmission cell, sealed with two Teflon stopcocks, and then transported to an external IR spectrometer within a sealed ziplock plastic bag under dinitrogen. The cell was removed from the bag and the sample immediately analysed by IR spectroscopy, background correcting for C_6H_5F solvent. A very low intensity $v(N_2)$ band was observed at 2290 cm^{-1} and this assignment was supported by (a) exposure of the sample to air, which resulted in complete conversion of **1-N$_2$** into **1-OH$_2$** within 5 seconds, (b) analysis by ATR-IR spectroscopy, where the sample was added directly onto the reflectance crystal of a spectrometer housed within the dinitrogen filled glovebox using rigorously dried glassware and allowed to evaporate; and (c) computational analysis.

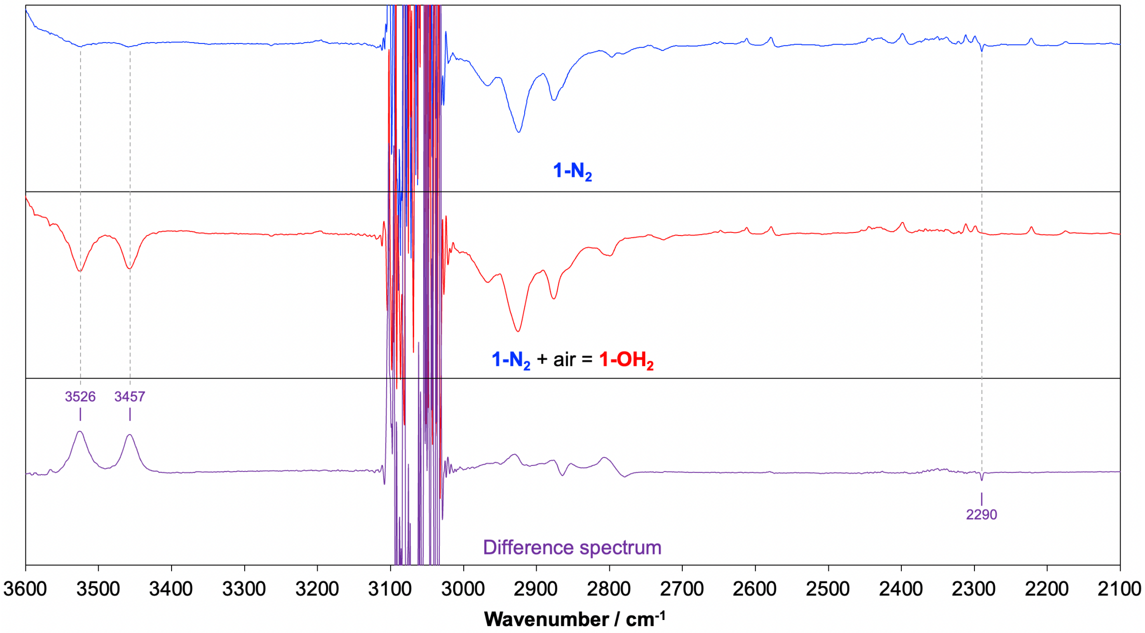

Figure S35. IR spectrum of **1-N$_2$** in C_6H_5F solution

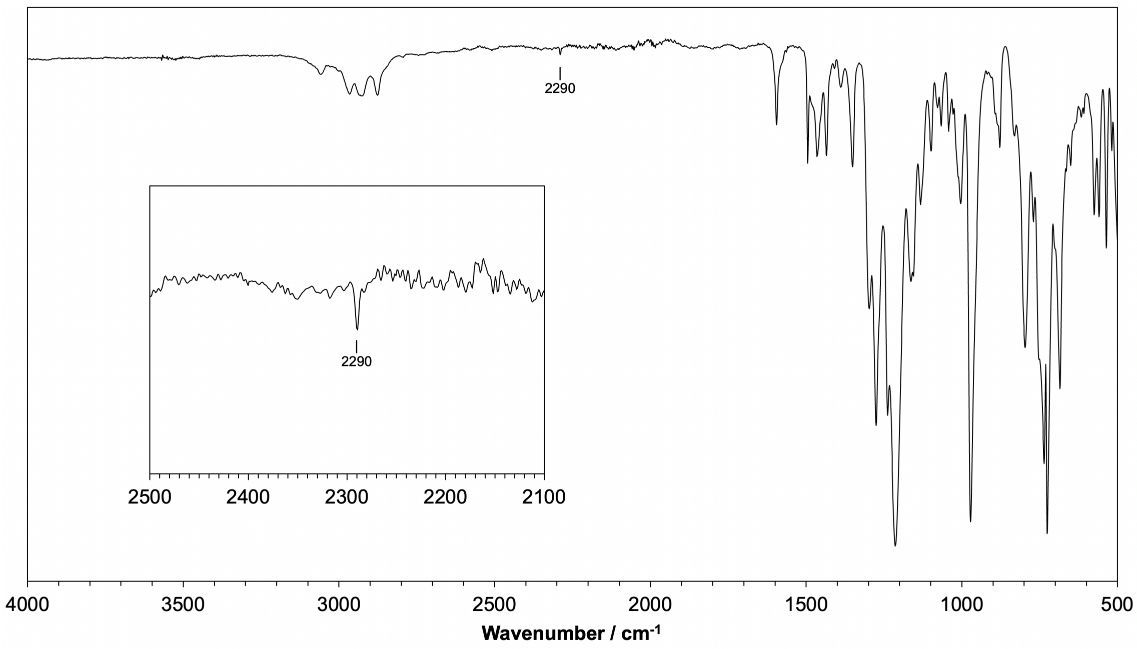

Figure S36. ATR-IR spectrum of **1-N_2**

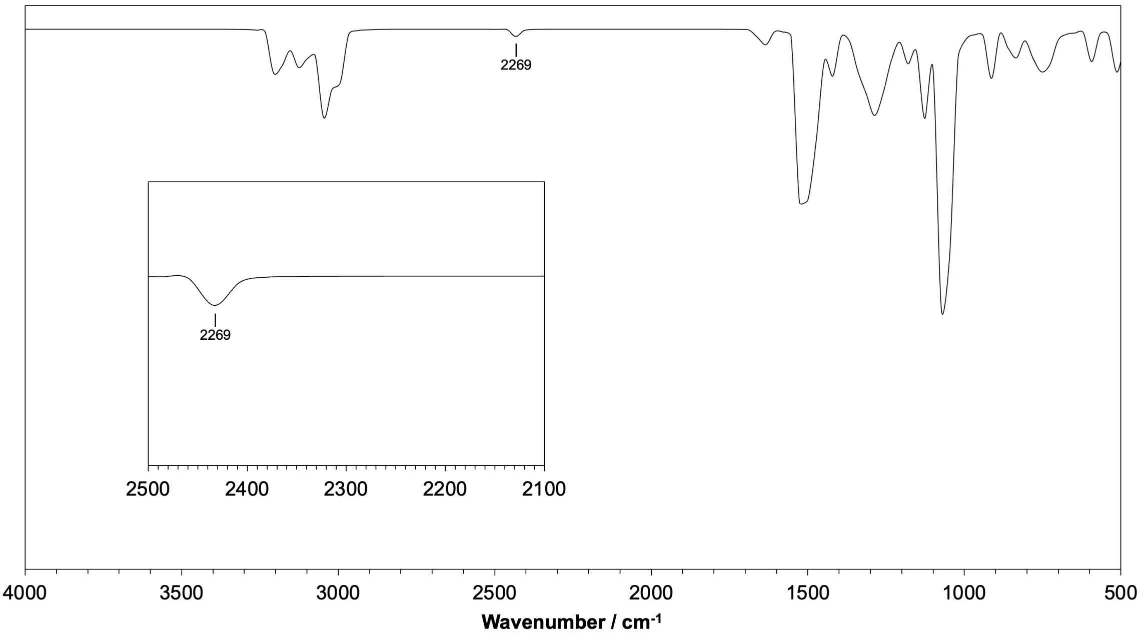

Figure S37. Calculated IR spectrum of **1-N_2** at the B3LYP-D3(BJ)/TZ2P-ZORA//B3LYP-D3(BJ)/def2-SVP level of theory. Data has been rescaled to match the experimental value for free N_2 (2330 cm^{-1}) at this level of theory, leading to a scaling factor of 0.936.

5. Crystallography

Data were collected on a Rigaku Oxford Diffraction SuperNova AtlasS2 CCD diffractometer using graphite monochromated Mo Kα (λ = 0.71073 Å) or CuKα (λ = 1.54184 Å) radiation and an Oxford Cryosystems *N*-HeliX low temperature device [150(2) K]. Data were collected and reduced using CrysAlisPro and refined using SHELXT,[13] through the Olex2 interface.[14]

Both complexes are isomorphous and crystallise with 1.5 equivalents of hexane in the low symmetry *P*-1 space group. All non-hydrogen atoms were refined anisotropically. Hydrogen atoms were placed in calculated positions and refined using the riding model. Disorder of two of the cation $OCH_2CH_2CH_3$ substituents was treated by modelling the propyl chains over two sites and restraining their geometry. Disorder of three of the anion $OC(CF_3)_3$ substituents was treated by modelling the CF_3 groups over two sites and restraining their geometry. Disorder of both hexane solvent molecules was treated by modelling them over two sites and restraining their geometry. Restraints to thermal parameters were applied where necessary in order to maintain sensible values.

Full details about the collection, solution, and refinement are documented in CIF format, which have been deposited with the Cambridge Crystallographic Data Centre under CCDC 2225292 (**1-OH$_2$**) and 2225293 (**1-N$_2$**).

6. Computational details

The geometry optimizations followed by the harmonic frequency calculations were carried out at the B3LYP-D3(BJ)/def2-SVP or PBE-D3(BJ)/def2-SVP level of theory using the Gaussian 16 suit of programs.[15,16] Effective core potentials were used for the 28 core electrons of Rh. Superfine integration grid is considered for all cases. Cartesian coordinates at the latter level of theory are provided in XYZ format.

The energy decomposition analysis (EDA)[17] in combination with the natural orbital for chemical valence (NOCV)[18] method was performed at the PBE-D3(BJ)-ZORA/TZ2P//PBE-D3(BJ)/def2-SVP level using the ADF (2018.105) program package.[19,20] The zeroth-order regular approximation (ZORA)[21] was used to include scalar relativistic effects. All electrons were considered in the computations. In the EDA method, the interaction energy (ΔE_{int}) between two prepared fragments is divided into three energy terms, *viz.*, the electrostatic interaction energy ($\Delta E_{\mathrm{elstat}}$), which represents the quasiclassical electrostatic interaction between the unperturbed charge distributions of the prepared fragments, the Pauli repulsion ($\Delta E_{\mathrm{Pauli}}$), which is the energy change associated with the transformation from the superposition of the unperturbed electron densities of the isolated fragments to the wavefunction that properly obeys the Pauli principle through explicit antisymmetrization and renormalization of the product wavefunction, and the orbital interaction energy (ΔE_{orb}), which is originated from the mixing of orbitals, charge transfer and polarization between the isolated fragments. Use of DFT-D3 with the Becke-Johnson type damping function (BJ) gives

additional dispersion interaction energy (ΔE_{int}(disp)) between two interacting fragments. Therefore, the total interaction energy (ΔE_{int}) between two fragments can be defined as:

$$\Delta E_{int} = \Delta E_{int}(\mathrm{disp}) + \Delta E_{int}(\mathrm{elec}) \quad (1)$$

$$\Delta E_{int}(\mathrm{elec}) = \Delta E_{elstat} + \Delta E_{Pauli} + \Delta E_{orb} \quad (2)$$

The EDA-NOCV combination allows the partition of ΔE_{orb} into pairwise contributions of the orbital interactions, which gives important information about bonding. The deformation density $\Delta\rho_k(r)$ which originates from the mixing of the NOCVs ψ_k(r) and ψ_{-k}(r), resulting from diagonalizing the deformation density matrix, gives the charge flow due to orbital interactions (equation 3), and the corresponding ΔE_k^{orb} reflects the amount of orbital interaction energy coming from such interaction which sums to the total orbital energy (equation 4).

$$\Delta\rho_{orb}(r) = \sum_k \Delta\rho_k(r) = \sum_K^{N/2} \nu_k \left[-\psi_{-k}^2(r) + \psi_k^2(r)\right] \quad (3)$$

$$\Delta E_{orb} = \sum_k \Delta E_k^{orb} = \sum_{k=1}^{N/2} \nu_k \left[-F_{-k}^{TS} + F_k^{TS}\right] \quad (4)$$

For further information about this method and its application, readers are referred to the related reviews.[22]

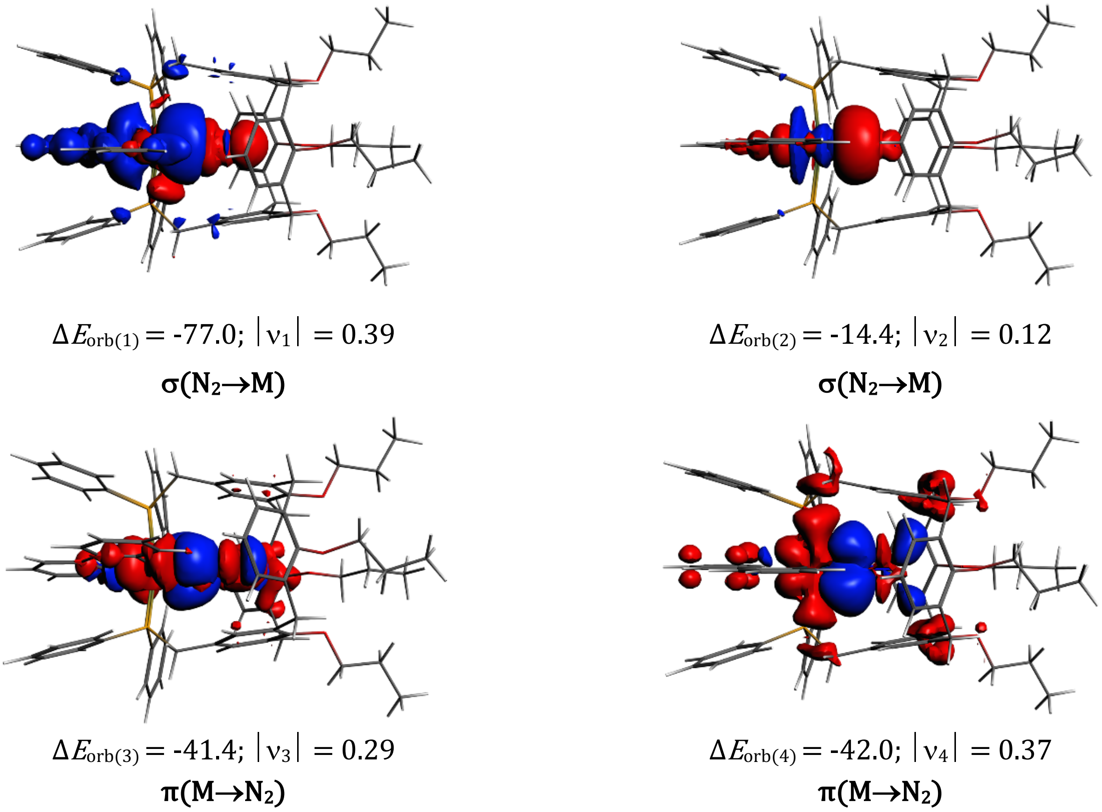

Figure S38. Deformation densities in **1-N$_2$**. ΔE_{orb} values are given in kJ·mol^{-1}. Charge flow is from red to blue and ν represents the charge eigenvalues and the iso-surface value is 0.0001 au.

Table S1. EDA-NOCV of substrate binding in **1-L** and **2-L** (L = H_2O, DCM, N_2).[a]

Energy	1-OH₂		2-OH₂		1-DCM		2-DCM		1-N₂		2-N₂	
ΔE_{int}	-125.0		-99.4		-84.6		-87.7		-93.2		-84.2	
$\Delta E_{int}(disp)$[b]	-32.4	(25.9%)	-22.8	(22.9%)	-58.8	(69.5%)	-50.1	(57.1%)	-41.1	(44.1%)	-24.6	(29.2%)
$\Delta E_{int}(elec)$[b]	-92.6	(74.1%)	-76.6	(77.1%)	-25.8	(30.5%)	-37.6	(42.9%)	-52.1	(55.9%)	-59.6	(70.8%)
ΔE_{Pauli}	267.6		225.2		250.0		203.0		359.0		368.0	
ΔE_{elstat}[c]	-220.4	(61.2%)	-190.9	(63.2%)	-159.4	(57.8%)	-141.8	(58.9%)	-224.2	(54.5%)	-231.2	(54.1%)
ΔE_{orb}[c]	-139.8	(38.8%)	-111.0	(36.8%)	-116.5	(42.2%)	-98.9	(41.1%)	-186.9	(45.5%)	-196.4	(45.9%)
$\Delta E_{L\rightarrow M}$[d]	-78.9	(56.4%)	-69.2	(62.3%)	-48.5	(41.6%)	-47.8	(48.3%)	-91.4	(48.9%)	-139.8[e]	(71.2%)
$\Delta E_{M\rightarrow L}$[d]					-19.8	(17.0%)	-20.3	(20.5%)	-83.4	(44.6%)	-48.1	(24.5%)
$\Delta E_{OH\cdots\pi}$[d,f]	-35.9	(25.7%)	-23.2	(20.9%)								
$\Delta E_{orb(rest)}$[d]	-25.0	(17.9%)	-18.6	(16.8%)	-48.2	(41.4%)	-30.8	(31.1%)	-12.1	(6.5%)	-8.5	(4.3%)
ΔE_{prep}	6.1		9.8		50.7		16.4		11.3		15.6	
E_{bond} ($-D_e$)	-118.9		-89.6		-33.9		-71.3		-81.9		-68.6	

[a] PBE-D3(BJ)/TZ2P-ZORA on structures at the PBE-D3(BJ)/def2-SVP level. Energy values in kJ·mol^{-1}. [b] The values in parentheses are the percentage contributions to the total interaction energy. [c] The values in parentheses are the percentage contributions to the total attractive interactions (ΔE_{elstat} + ΔE_{orb}). [d] The values in parentheses are the percentage contributions to the total orbital term, ΔE_{orb} resulting from analysis of the NOCV results. [e] L→M and M→L interactions cannot clearly be distinguished (see Figure S39). [f] Interactions unique to **1-OH₂** and **2-OH₂**.

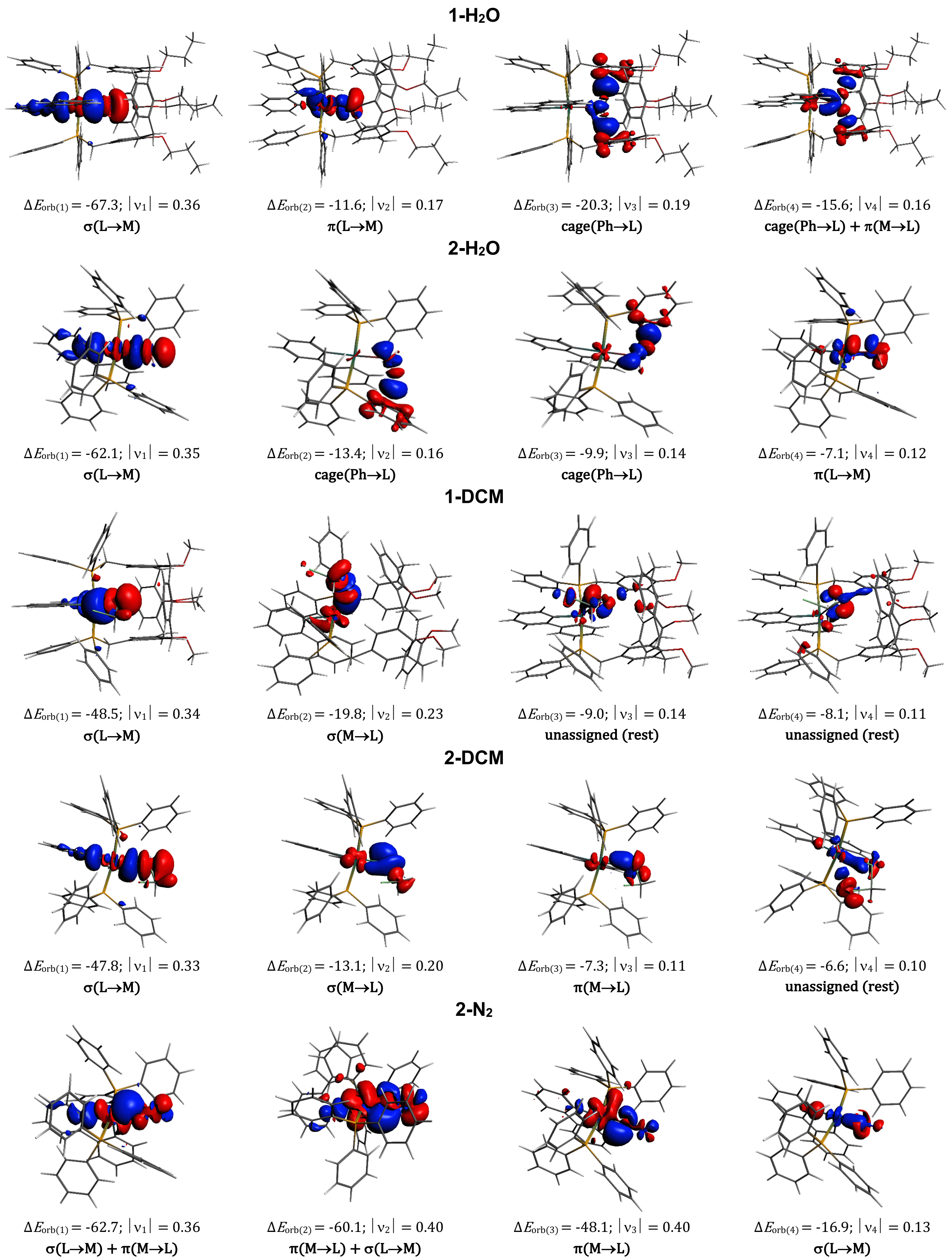

Figure S39. Deformation densities in **1-L** (L = OH_2, DCM) and **2-L** (L = OH_2, DCM, N_2). ΔE_{orb} values are given in kJ·mol^{-1}. Charge flow is from red to blue and ν represents the charge eigenvalues and the iso-surface value is 0.0005 au.

7. References

[1] S. D. Pike, M. R. Crimmin and A. B. Chaplin, *Chem. Commun.*, 2017, **53**, 3615–3633.

[2] R. C. Knighton, J. Emerson-King, J. P. Rourke, C. A. Ohlin and A. B. Chaplin, *Chem. Eur. J.*, 2018, **24**, 4927–4938.

[3] C. N. Iverson and W. D. Jones, *Organometallics*, 2001, **20**, 5745–5750.

[4] X. Fang, B. L. Scott, J. G. Watkin, C. A. G. Carter and G. J. Kubas, *Inorg. Chim. Acta.*, 2001, **317**, 276–281.

[5] P. S. Pregosin, *NMR in Organometallic Chemistry*, Wiley-VCH, 2012, pp. 251–254.

[6] R. Streck and A. J. Barnes, *Spectrochim. Acta A*, 1999, **55**, 1049–1057.

[7] C. D. Gutsche and J. A. Levine, *J. Am. Chem. Soc.*, 1982, **104**, 2652–2653.

[8] A. Dondoni, C. Ghiglione, A. Marra and M. Scoponi, *Macromol. Chem. Phys.*, 1999, **86**, 77–86.

[9] M. Larsen and M. Jørgensen, *J. Org. Chem.*, 1996, **61**, 6651–6655.

[10] M. H. Düker, F. Kutter, T. Dülcks and V. A. Azov, *Supramol. Chem.*, 2014, **26**, 552–560.

[11] A. Marra, M.-C. Scherrmann, A. Dondoni, R. Ungaro, A. Casnati and P. Minari, *Angew. Chem. Int. Ed.*, 1995, **33**, 2479–2481.

[12] S. Donovan-Mtunzi, R. L. Richards and J. Mason, *J. Chem.* Soc.*, Dalton Trans.* 1984, 2429–2433

[13] G. M. Sheldrick. *Acta Cryst.*, 2015, **71**, 3–8.

[14] O.V. Dolomanov, L. J. Bourhis, R. J. Gildea, J. A. K. Howard and H. Puschmann, *J. Appl. Cryst.*, 2009, **42**, 339–341.

[15] (a) A. D. Becke, *Phys. Rev. A* 1988, **38**, 3098–3100; (b) C. Lee, W. Yang and R. G. Parr, *Phys. Rev. B.* 1988, **37**, 785–789; (c) J. P. Perdew, K. Burke and M. Ernzerhof, *Phys. Rev. Lett.*, 1996, **77**, 3865-3868; (d) S. Grimme, J. Antony, S. Ehrlich and H. Krieg, *J. Chem. Phys.*, 2010, **132**, 154104; (e) F. Weigend and R. Ahlrichs, *Phys. Chem. Chem. Phys.*, 2005, **7**, 3297-3305; (f) F. Weigend, *Phys. Chem. Chem. Phys*. 2006, **8**, 1057-1065.

[16] Gaussian 16, Revision A.03, M. J. Frisch, G. W. Trucks, H. B. Schlegel, G. E. Scuseria, M. A. Robb, J. R. Cheeseman, G. Scalmani, V. Barone, G. A. Petersson, H. Nakatsuji, X. Li, M. Caricato, A. V. Marenich, J. Bloino, B. G. Janesko, R. Gomperts, B. Mennucci, H. P. Hratchian, J. V. Ortiz, A. F. Izmaylov, J. L. Sonnenberg, D. Williams-Young, F. Ding, F. Lipparini, F. Egidi, J. Goings, B. Peng, A. Petrone, T. Henderson, D. Ranasinghe, V. G. Zakrzewski, J. Gao, N. Rega, G. Zheng, W. Liang, M. Hada, M. Ehara, K. Toyota, R. Fukuda, J. Hasegawa, M. Ishida, T. Nakajima, Y. Honda, O. Kitao, H. Nakai, T. Vreven, K. Throssell, J. A. Montgomery, Jr., J. E. Peralta, F. Ogliaro, M. J. Bearpark, J. J. Heyd, E. N. Brothers, K. N. Kudin, V. N. Staroverov, T. A. Keith, R. Kobayashi, J. Normand, K. Raghavachari, A. P. Rendell, J. C. Burant, S. S. Iyengar, J. Tomasi, M. Cossi, J. M. Millam, M. Klene, C. Adamo, R. Cammi, J. W. Ochterski, R. L. Martin, K. Morokuma, O. Farkas, J. B. Foresman, and D. J. Fox, Gaussian, Inc., Wallingford CT, 2016.

[17] T. Ziegler and A. Rauk, *Theor. Chim. Acta*, 1977, **46**, 1-10.

[18] M. Mitoraj and A. Michalak, *Organometallics* 2007, **26**, 6576-6580.

[19] ADF2018, SCM, Theoretical Chemistry, Vrije Universiteit, Amsterdam, The Netherlands, http://www.scm.com (accessed 15/12/2022).

[20] G. Te Velde, F. M. Bickelhaupt, E. J. Baerends, C. F. Guerra, S. J. A. van Gisbergen, J. G. Snijders and T. Ziegler, *J. Comput. Chem.*, 2001, **22**, 931-967.

[21] E. van Lenthe, A. E. Ehlers and E. J. Baerends, *J. Chem. Phys.*, 1999, **110**, 8943-8953.

[22] (a) G. Frenking and F. M. Bickelhaupt, in *The Chemical Bond: Fundamental Aspects of Chemical Bonding*, ed. G. Frenking and S. Shaik, Wiley-VCH, 2014, ch. 4, pp. 121–157; (b) L. Zhao, M. von Hopffgarten, D. M. Andrada, and G. Frenking, *WIREs Comput. Mol. Sci.*, 2018, **8**, e1345; (c) L. Zhao, M. Hermann, W. H. E. Schwarz, and G. Frenking, *Nat. Rev. Chem.*, 2019, **3**, 48-63.
